# Polymers of Intrinsic Microporosity as Organic Cathodes for Fast‐Charging Lithium‐Ion Batteries

**DOI:** 10.1002/smsc.70364

**Published:** 2026-08-13

**Authors:** Eva Vásquez‐Barillas, Ani N. Davis, A M Mahmudul Hasan, Amrit Kaur, Swapnanil Goswami, Kiana A. Treaster, Kausturi Parui, John D. Langhout, Rachel H. Bianculli, Joshua D. Marquez, Gabriel Carson, Austin M. Evans, Megan M. Butala

**Affiliations:** ^1^ George and Josephine Butler Polymer Laboratory Department of Chemistry University of Florida Gainesville Florida USA; ^2^ Department of Materials Science and Engineering University of Florida Gainesville Florida USA

**Keywords:** charge‐storage mechanisms, fast charging, lithium‐ion battery, organic electrode, polymers of intrinsic microporosity, rate capability

## Abstract

Developing fast‐charging electrodes requires understanding how polymer architecture governs ion‐transport, redox‐site accessibility, and charge‐storage mechanism. While porosity improves electrochemical performance, its role at fast‐charging conditions remains unclear. We investigate the effects of porosity by comparing polymers with a naphthalene diimide (NDI) redox‐active unit and two contorted monomers (triptycene‐like [tryp] and spirobisindane [spiro]) to a nonporous model system (NDI‐model). In galvanostatic cycling, NDI‐model exhibits low accessible capacity even at slow rates (25.8 mAh g^−1^ at C/10), indicating limited ion transport, related to its low surface area (35 m^2^ g^−1^). NDI‐spiro exhibits a superior performance, retaining 74% of its C/10 capacity at 20C. Cyclic voltammetry reveals a surface‐controlled charge‐storage mechanism for NDI‐spiro, attributed to its higher surface area (273 m^2^ g^−1^), which allows greater accessibility of redox sites. In contrast, NDI‐tryp achieves high capacity at slow rates (50.4 mAh g^−1^ at C/10), but suffers from diffusion limitations and poor rate capability, related with its diffusion‐controlled charge‐storage mechanism. Extending this study to spiro‐based polymers with other redox active units, perylene diimide (PDI) and benzenediimide (BDI), shows that redox‐unit identity affects kinetics and rate capability, shifting the charge‐storage mechanism from surface‐controlled to a diffusion‐controlled. These findings establish structure–property relationships for designing high‐performance fast‐charging organic battery electrodes.

## Graphical abstract

Polymer architecture and redox‐unit identity govern ion accessibility, charge‐storage mechanism, and rate capability in organic lithium‐ion battery cathodes, revealing molecular design principles for next‐generation fast‐charging organic electrodes.

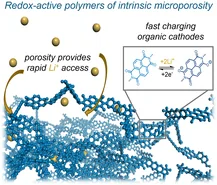

## 1 Introduction

The global transition to electrified transportation, portable electronics, and renewable energy integration increasingly depends on fast‐charging energy storage technologies [1, 3]. However, energy storage systems capable of fast cycling remain fundamentally limited by the use of conventional metal oxide cathodes in Li‐ion batteries. These electrodes also rely on expensive and scarce transition metals, such as Ni and Co, which are expected to face availability challenges for large‐scale production [4, 5]. Additionally, although inorganic electrodes like Li(Ni,Mn,Co)O_2_ and LiFePO_4_ have favorable performance in metrics such as high energy density, their dense structures rely on ion intercalation via solid‐state diffusion, which imposes kinetic limitations [6, 8]. In an intercalation mechanism, Li ions navigate through the crystal lattice, facing diffusion bottlenecks that can limit rate capabilities (i.e., performance under fast cycling conditions) [9, 10]. Considering these limitations, organic materials are attractive as alternative electrodes for fast‐charging metal‐ion batteries because they can be systematically synthetically modified, which can be leveraged to improve kinetics, and their carbon‐based composition, which relies on more abundant elements [11, 12].

Organic electrodes offer structurally tunable frameworks, accessible redox‐active centers, and greater flexibility than inorganic materials, enabling more efficient ion transport. In particular, polymeric systems provide unique design opportunities by enabling control over supramolecular interactions, which can lead to high‐performance electrodes [13, 16]. Specifically, porosity has emerged as a key design principle for improving ion transport kinetics by enhancing ion accessibility and transport pathways [17, 19], with porous organic polymers offering an especially exciting combination of high energy density, high reversible capacity, and cycling stability [20, 21]. Within this context, polymers of intrinsic microporosity (PIMs) are a class of materials characterized by a highly contorted backbone that generates abundant microporous channels, a feature that has been well documented in porous membranes for gas separation, heterogeneous catalysis, gas separation, and hydrogen storage [22, 23]. Previous work by Evans and coworkers demonstrated that introducing backbone contortion through spirobisindane (spiro) units enables rapid redox switching (*t*_95_ ≈ 1 s) with high optical contrast and high coloration efficiency in electrochromic devices. This work showed that the balance between surface area and electronic conductivity governs kinetics in porous mixed ionic–electronic systems, which is a desirable property of polymers for fast‐charging lithium‐ion batteries [24, 25]. Similarly, Song and coworkers developed a solution‐processable PIM with redox activity, which showed that installing a contorted unit in the rigid backbone enhances Li‐ion diffusion and capacity retention during cycling [26]. These observations are related to the high surface area and sub‐nanometer pores of PIMs, which shorten diffusion distances to redox‐active sites and mitigate polarization [27, 28].

However, there is still a need for in‐depth understanding of structure‐property relationships of ion transport and charge‐storage mechanisms in PIMs, including relationships between the porosity, provided by the rigid contorted backbone, and the redox‐active unit. In electrodes, redox processes can occur at a material's surface through surface‐controlled charge‐storage mechanisms or in the bulk through diffusion‐controlled mechanisms [29, 30]. To assess a material for high‐power applications, battery performance at fast cycling is evaluated as rate capability, or how much of the capacity at slow rates is retained at fast cycling rates, which requires transport of both ions and electrons [31]. In this context, previous work by McKeown and coworkers shows that in porous organic electrodes, surface‐controlled charge‐storage mechanisms enhance access to redox‐active sites, providing accessible redox‐active sites under fast charge/discharge cycling conditions [32]. Therefore, tailoring electrode materials to optimize both ionic and electronic transport is critical to maintaining a high accessible capacity at rapid charge/discharge performance.

**FIGURE 1:**
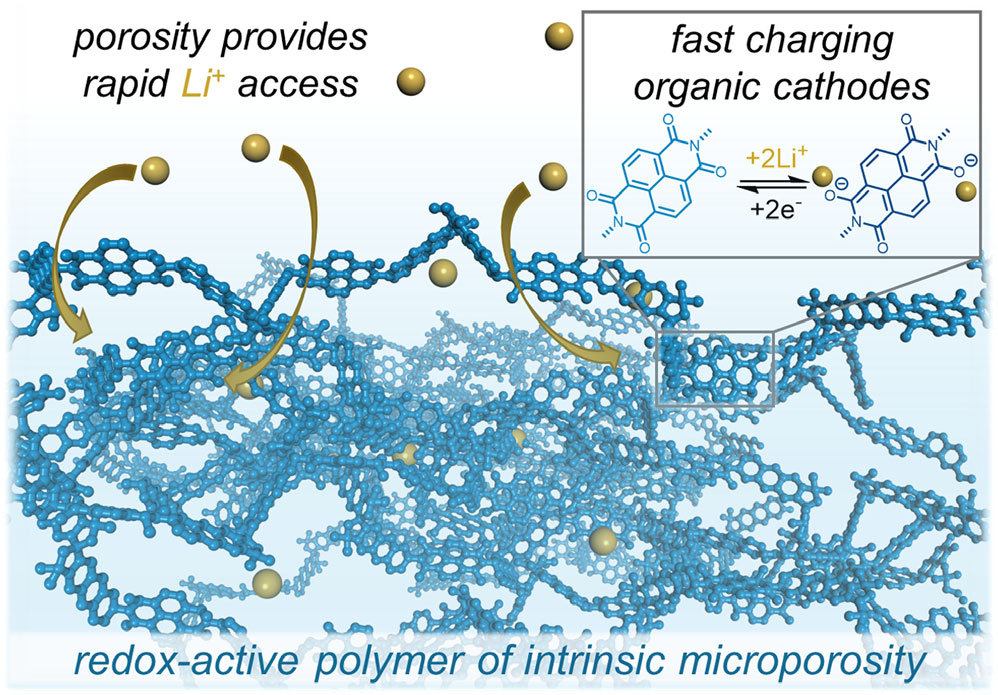
Illustrative depiction of a polymer of intrinsic microporosity electrode for Li‐ion batteries for fast charging.

Given the demonstrated promise of PIMs for accessible Li‐ion transport, we use these as a platform to systematically probe the effects of porosity, surface area, and redox identity on fast‐charging capabilities. We modify the architecture of redox‐active polymers by using different linkers, including two contorted linkers previously shown to induce porosity [22, 26, 33] and a model linker that allows efficient packing and forms a nonporous polymer (Figure 1). The spirobisindane linker (spiro) exhibits a point of contortion that enables rigidity and inefficient packing in the polymer chain [22]. In addition to the previously studied contorted linker, spiro, we use the naphthopleiadene linker, developed by McKeown and coworkers, which resembles a triptycene‐like unit (tryp) and introduces contortion through its rigid three‐dimensional architecture [33]. Using the contorted tryp‐linker, we synthetized a redox‐active polymer that is studied here for the first time as an organic electrode.

Our results show that polymers combining porosity and high surface area provide continuous transport pathways for ion accessibility, promoting fast charging, whereas polymers lacking these properties exhibit reduced utilization of redox‐active sites under fast cycling rates. Further, we probe how variations in the redox‐active unit alter the charge‐storage mechanism and, consequently, performance under fast cycling conditions by comparing different redox‐active units within the same contorted unit (spiro). By evaluating the effects of contorted linkers and redox‐active units on polymer architecture and cycling behavior, this work establishes design principles to achieve fast‐charging cathodes by correlating microporosity with electrochemical kinetics and high‐rate performance capabilities. To assess the utility of microporosity as a design strategy for fast‐charging Li‐ion batteries, we assessed the battery cycling behavior of these polymers in half‐cells (i.e., with a Li metal anode). By comparing the battery cycling behavior and ion transport mechanisms of these materials, we identify effects of porosity, surface area, and redox mechanism on the performance of organic electrodes for fast‐charging Li‐ion batteries.

## 2 Results and Discussion

### 2.1 Synthesis and Characterization of Polymers

**FIGURE 2:**
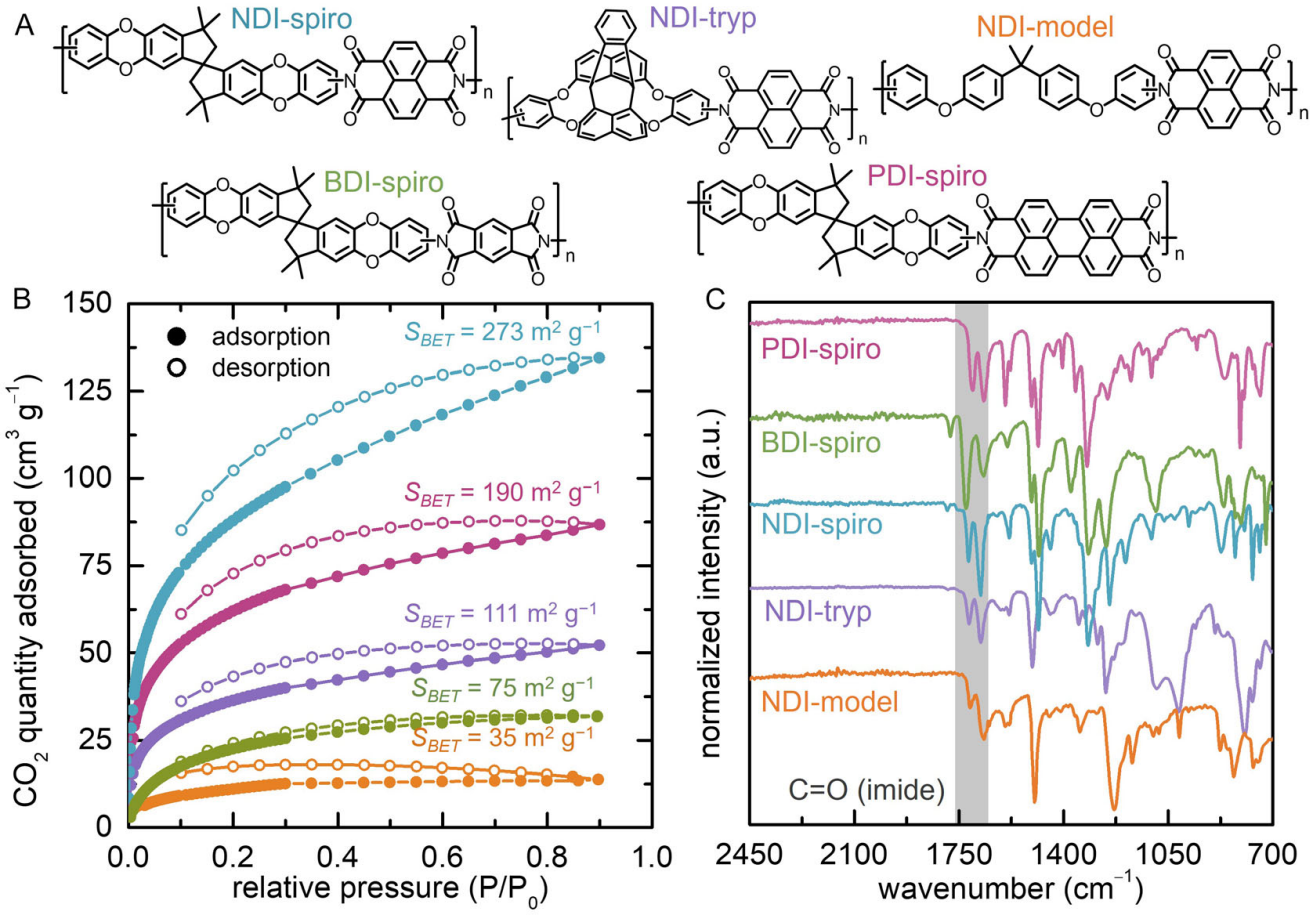
(A) Chemical structures of redox‐active polymers of intrinsic microporosity (PDI‐spiro, BDI‐spiro, NDI‐spiro, and NDI‐tryp) and model system (NDI‐model). (B) CO_2_ isotherms measured at 195 K of NDI‐spiro, PDI‐spiro, BDI‐spiro, NDI‐tryp, and NDI‐model with their respective surface areas (*S*_BET_). (C) FTIR spectra of the synthesized polymers of intrinsic microporosity (NDI‐spiro, PDI‐spiro, BDI‐spiro, NDI‐tryp, and NDI‐model) highlighting the imide C=O stretch between 1650 and 1690 cm^−1^.

To examine how intrinsic microporosity modulates redox behavior, a series of redox‐active polymers was synthesized by combining a redox‐active unit with amine‐functionalized linkers. The redox‐active unit imparts redox functionality, while contorted linkers disrupt efficient chain packing within the polymer by introducing backbone rigidity and generating intrinsic microporosity (Figure 2A). For these studies, we selected naphthalene diimide (NDI) as one redox linker, as it is a well‐studied redox‐active unit in Li‐ion battery electrodes [34]. To compare the effect of the contorted unit in battery performance, we synthetized spiro‐based and tryp‐based redox active polymers. The linkers studied in this work vary in the structure of the contorted unit, which were intended to affect the porosity. As a control, a noncontorted bisphenol‐based linker was used as a more flexible model system that would enable efficient chain packing, resulting in a polymer lacking microporosity. To further study the effect of the redox‐active unit, we synthesized spiro‐based PIMs by varying the redox‐active unit, specifically perylene diimide (PDI) and benzenediimide (BDI), in addition to NDI.

The materials were synthesized via condensation polymerization of a dianhydride redox‐active unit and an amine‐functionalized linker to give diimide‐linked PIMs (NDI, PDI, or BDI), following a previously reported synthetic procedure (see Supporting Information for the synthesis details) [24, 35]. In the starting materials, characteristic infrared spectroscopy features corresponding to the anhydride and amine groups are evident (Figures S11–S15). After the polymerization reaction, Fourier‐transform infrared (FTIR) spectra show a new strong band between 1650 and 1690 cm^−1^, corresponding to the imide C=O stretch (Figure 2C). Furthermore, the disappearance of the amine and anhydride vibration bands from the starting materials further supports the successful condensation reaction (Figures S11–S15), indicating that an imide bond was effectively formed in the final polymers.

Surface area was evaluated for all the materials by CO_2_ sorption isotherms collected at 198 K (Figure 2B), which is the most established protocol for evaluating porosity in PIMs [24, 26, 33, 36]. The NDI‐model system has a lower surface area of 35 m^2^ g^−^^1^, consistent with the absence of a contorted unit. For the spiro‐based PIMs (NDI‐spiro, PDI‐spiro, and BDI‐spiro), we observed surface areas of 273, 190, and 75 m^2^ g^−1^, respectively. NDI‐tryp has a surface area of 111 m^2^ g^−1^, due to its three‐dimensional structure that provides rigidity to the polymer chain. Among the spiro‐based polymers, BDI‐spiro exhibits the lowest surface area (75 m^2^ g^−1^), which we attribute to the smaller and less sterically demanding benzene diimide unit, which provides less backbone disruption relative to the larger NDI and PDI cores. PDI‐spiro achieves the second‐highest surface area (190 m^2^ g^−1^), consistent with the greater steric bulk and rigidity of the perylene core relative to BDI, which more effectively disrupts chain packing.

### 2.2 Effect of Microporosity on Rate‐Dependent Electrochemical Behavior

To evaluate how porosity influences electrochemical performance, we analyzed cycling behavior and ion accessibility using galvanostatic cycling with potential limitations (GCPLs) in half‐cells (vs. Li metal) at varying rates, using gravimetric capacity and rate capability to understand polymer behavior. By taking the derivative of these cycling data, differential capacity (dQ/dV) plots can be generated and highlight the voltages at which redox processes occur, enabling assessment of ion accessibility, kinetics, and stability [37].

**FIGURE 3:**
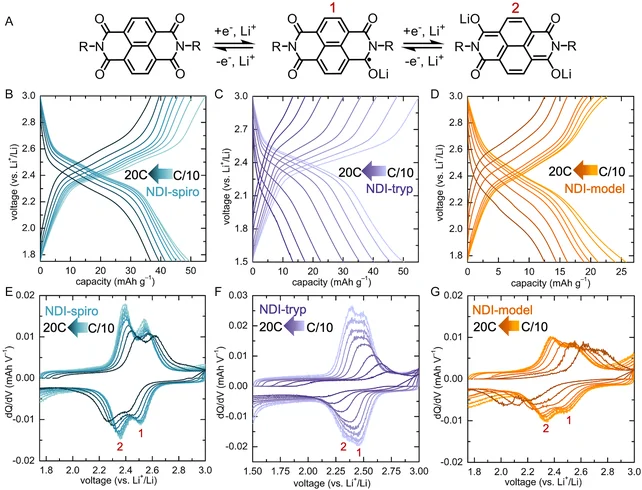
(A) Redox processes occurring in NDI‐based electrodes during electrochemical lithiation and delithiation. Galvanostatic charge–discharge profiles at different cycling rates of (B) NDI‐spiro, (C) NDI‐tryp, and (D) NDI‐model, as well as differential capacity plots at different cycling rates of (E) NDI‐spiro, (F) NDI‐tryp, and (G) NDI‐model. The 10th cycle at each rate is displayed for each material at rates of C/10, C/5, C/2, 1C, 2C, 5C, 10C, and 20C.

NDI‐based materials exhibit two redox features corresponding to lithiation of NDI (Figure 3A) [14], which are observable in the GCPL and dQ/dV plots. Under slow cycling (C/10), NDI‐spiro and NDI‐tryp achieved 71% (50.4 mAh g^−1^) and 77% (50.3 mAh g^−1^) of their theoretical capacities (71.4 and 64.7 mAh g^−1^, respectively) (Figures S16A and S17A). Both materials exhibit well‐defined plateaus under slow cycling, indicating efficient ion accessibility under nondemanding (i.e., slow rate) conditions, related to the accessible surface areas of these materials. In contrast, NDI‐model shows poorly defined sloping plateaus and achieves only 31% of its theoretical capacity (25.8 mAh g^−1^ of 83.4 mAh g^−1^) (Figure S18A). This is indicative of poor kinetics and limited ion transport, which we attribute to a lack of microporosity. With increasing rate (from C/10 to 20C), NDI‐spiro retains its plateaus’ shapes, consistent with efficient ion transport (Figure 3B). NDI‐model exhibits increasingly steep plateaus as the rate increases, indicating transport limitations (Figure 3D). NDI‐tryp shows a significant loss of feature definition at higher cycling rates, suggesting reduced ion accessibility and possibly a different charge‐storage mechanisms than that of NDI‐spiro, despite its high surface area (Figure 3C).

Rate‐dependent shifts in redox potential can be observed from dQ/dV plots derived from GCPL cycling data, where NDI‐spiro maintains relatively symmetric charge/discharge features, with only modest voltage shifts at high rates, indicating limited polarization and favorable kinetics (Figure 3E). NDI‐tryp behaves similarly to NDI‐spiro at C/10, showing two redox peaks, but as the cycling rate increases, the two distinct features merge into a single broad feature with higher overpotentials, indicating increased polarization and less efficient charge storage (Figure 3F). These findings highlight that the charge‐storage mechanism in NDI‐tryp is inefficient with increasing cycling rate. In contrast, NDI‐model shows large voltage shifts and significant peak broadening and merging, consistent with kinetic limitations and poor ion transport (Figure 3G).

**FIGURE 4:**
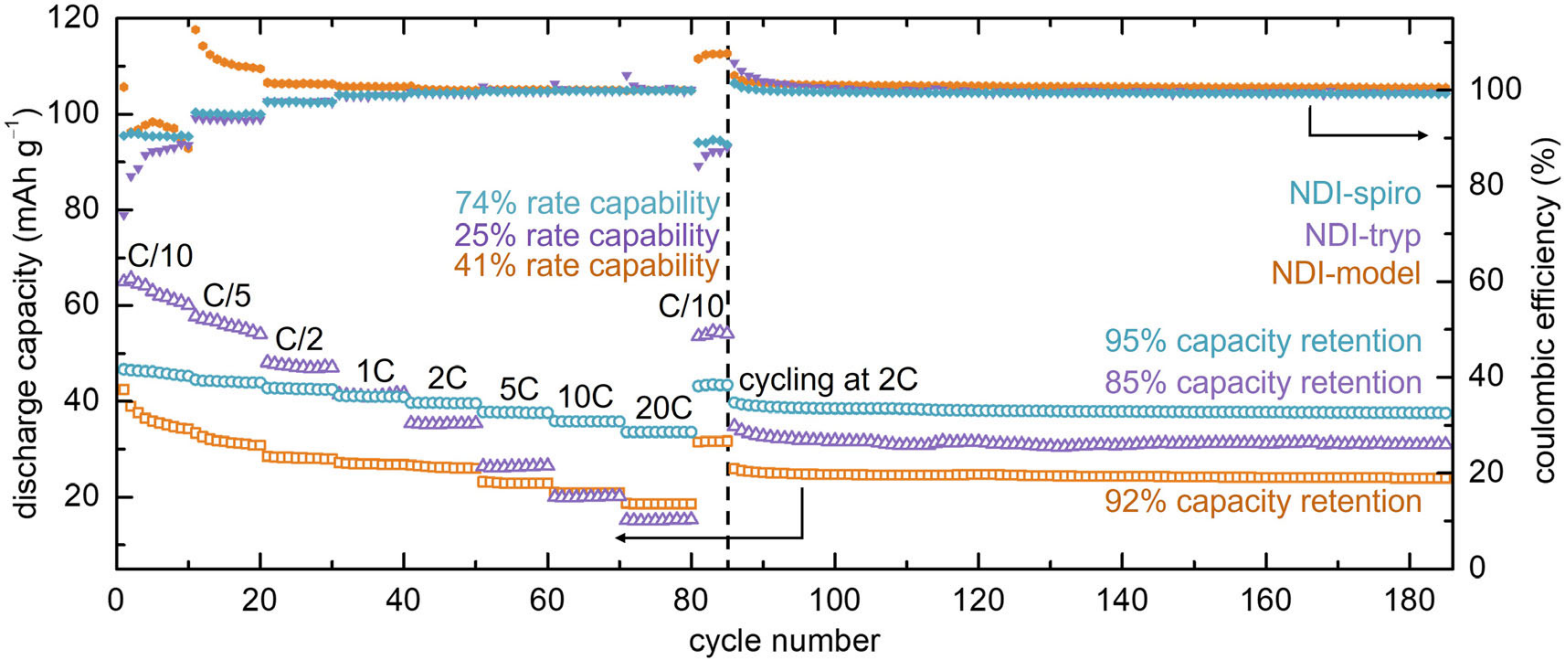
Average discharge capacities (bottom left) and Coulombic efficiencies (top left) of NDI‐spiro, NDI‐tryp, and NDI‐model during variable rate cycling (from C/10 to 20C). Average discharge capacities (bottom right) and Coulombic efficiencies (top right) during cycling at 2C for an additional 100 cycles. The capacities and Coulombic efficiency values at each cycle number are the average of three representative cells for each material (plotted with standard deviations in Figure S27).

The different rate capabilities are further reflected in the achievable capacities during variable rate cycling. The nonporous NDI‐model has a rate capability of 41%, with low capacities at all cycling rates (Figure 4, left panel). From C/10 to 20C, NDI‐spiro retains 74% of its slow rate capacity at fast rates, which is substantially higher than that of NDI‐tryp's rate capability of 25% (Figure 4, left panel). These observations are consistent with the qualitative analysis of the GCPL and dQ/dV plots of NDI‐tryp, which shows a high initial capacity, but has kinetic limitations that hinder ions’ access to the redox sites at faster cycling rates. Together, these results suggest that under slow conditions, the high surface of NDI‐tryp facilitates ion transport within the material. However, at higher rates, there is a different charge‐storage mechanism in NDI‐tryp, compared to that of NDI‐spiro (Figure 2C), which limits ion accessibility, resulting in poor rate performance. This kinetic limitation in NDI‐tryp is further supported by scanning electron microscopy (SEM) analysis of the pristine active materials and cast electrodes (Figures S31–S34), which reveals that NDI‐tryp consists of substantially larger and more agglomerated particles than NDI‐spiro and NDI‐model. Although NDI‐spiro and NDI‐tryp possess comparable microporous surface areas, the larger particle size and greater degree of agglomeration in NDI‐tryp likely reduce the accessible electrode–electrolyte interfacial area and increase ion transport distances, thereby suppressing surface‐controlled redox processes. These results suggest that fast‐charging performance is optimized not only by intrinsic microporosity but also by small, well‐dispersed particles that maximize ion accessibility and facilitate rapid charge transfer.

Cycling stability was assessed by extended cycling following variable rate tests. We find that NDI‐spiro, NDI‐tryp, and NDI‐model retain 95%, 85%, and 92%, respectively, of their capacity through 100 cycles at 2C (Figure 4, right panel). Possible mechanisms of degradation include irreversible charge‐storage processes, polymer degradation, and dissolution in the electrolyte [14, 38]. The capacity retention for NDI‐spiro indicates minimal degradation of charge‐storage abilities. However, NDI‐model and NDI‐tryp behave differently from NDI‐spiro. At a steady cycling rate of 2C, NDI‐model achieves 29% of its theoretical capacity and NDI‐tryp achieves 36%, while NDI‐spiro achieves 53%. This highlights that the microporous architecture of NDI‐spiro, along with its higher surface area, enables both efficient ion transport and excellent stability, providing superior rate capability without sacrificing long‐term cycling performance. In contrast, the nonporous, low‐surface‐area NDI‐model and NDI‐tryp suffer from limited ion diffusion and slower charge–transfer kinetics, resulting in lower capacities at fast cycling rates despite comparable structural stability.

All three materials exhibit high Coulombic efficiencies during variable rate and cycling stability experiments. During variable rate experiments, NDI‐spiro has a Coulombic efficiency of 97%, and NDI‐tryp and NDI‐model both exhibit a Coulombic efficiency of 100% (Figure 4, left). During cycling stability experiments, NDI‐spiro has a Coulombic efficiency of 99%, and NDI‐tryp and NDI‐model both have a Coulombic efficiency of 101% (Figure 4, right). These high Coulombic efficiency values indicate that the NDI‐redox sites undergo reversible electrochemical processes and that capacity loss due to irreversibility or parasitic side reactions is negligible.

### 2.3 Ion Transport Mechanisms in the Linker Series

To better understand the origins and differences in electrode performance, we used complementary electrochemical techniques to study kinetic limitations and charge‐storage mechanisms. Cyclic voltammetry (CV) provides information about redox accessibility and kinetic limitations by revealing how the current response evolves with scan rate and applied potential. In redox‐active polymers, peak symmetry, current retention at high scan rates, and peak broadening qualitatively reflect differences in ion transport and electronic conductivity [37, 39]. In CV, peak separation (hysteresis) between oxidation and reduction increases with scan rate as the current response lags behind voltage, indicating kinetic limitations [40]. When combined with electrochemical impedance spectroscopy (EIS) and distribution of relaxation times (DRT), these techniques provide a more complete picture of charge storage, ion transport, and the impact of kinetic bottlenecks on cycling [41]. Using CV, EIS, and DRT, we isolate the effect of porosity on electrochemical behavior by comparing NDI‐spiro with NDI‐tryp and NDI‐model.

**FIGURE 5:**
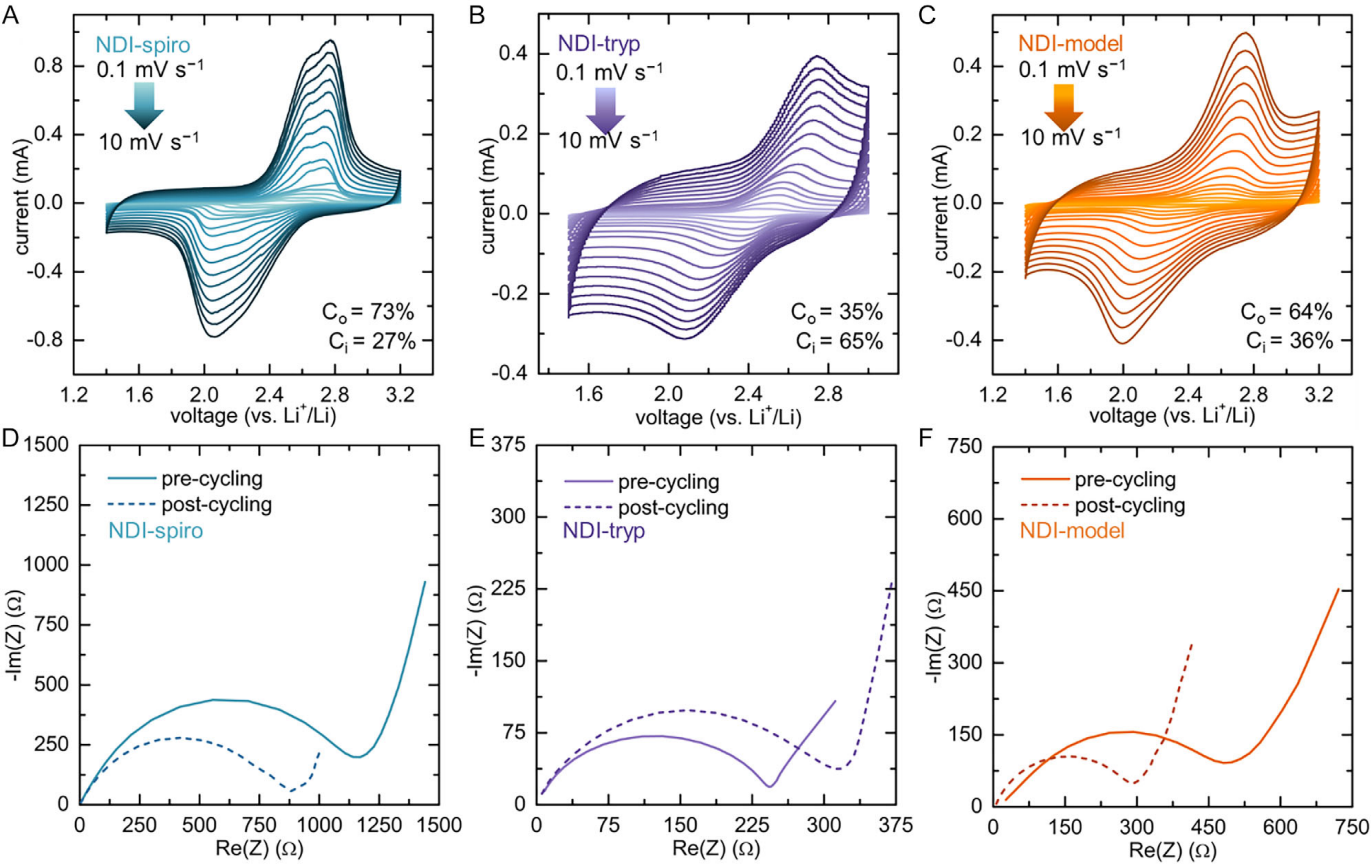
CV at scan rates from 0.1 to 10 mV s^−1^ for (A) NDI‐spiro, (B) NDI‐tryp, and (C) NDI‐model. The CVs were taken at 0.10, 0.25, 0.50, 0.75, 1.00, 1.5, 2.00, 3.00, 4.00, 5.00, 6.00, 7.00, 8.00, 9.00, and 10 mV s^−1^. Nyquist plots precycling (solid lines) and postcycling (dotted lines) of (D) NDI‐spiro, (E) NDI‐tryp, and (F) NDI‐model.

All three materials exhibit redox activity within a similar voltage window, confirming comparable redox chemistry of the NDI moiety (Figure 5A–C). NDI‐tryp shows two redox peaks with low hysteresis at slow scan rates (0.06 and 0.07 V), but broader features and significant peak shifting at higher scan rates, leading to increased hysteresis of Δ*E* = 0.64 V (Table S3). On the other hand, NDI‐spiro maintains a hysteresis of 0.10 V and 0.08 V at 0.1 mV s^−1^ and hysteresis of 0.29 and 0.37 V at 10 mV s^−1^, comparatively lower than that of NDI‐tryp (Table S3), indicating faster kinetics. The NDI‐model exhibits higher initial hysteresis (0.11 V, 0.15 V), which increases to 0.72 V at high scan rates, along with peak broadening and merging, consistent with slower kinetics of a nonporous, low‐surface‐area polymer.

To probe the charge‐storage mechanism, a Trasatti analysis was performed by plotting the integrated current against the reciprocal of the square root of the scan rate, which allows differentiating the relative contributions of surface‐controlled processes (or more kinetically accessible processes) and bulk, diffusion‐controlled processes to the charge storage [37, 42]. Accordingly, we calculated the percent of surface‐controlled (*C*_o_) and diffusion‐controlled (*C*_i_) charge storage (Table S2). This analysis showed that NDI‐spiro has a dominant surface‐controlled charge‐storage mechanism (*C*_o_ = 73%) (Figures 5A and S22). NDI‐model shows a storage mechanism similar to NDI‐spiro, with a dominant surface‐controlled charge‐storage mechanism (*C*_o_ = 64%) (Figures 5C and S24). The dominance of surface‐controlled contributions indicates that a large fraction of the redox processes occurs at particle or pore surfaces. However, because of the significantly larger hysteresis and peak merging at high scan rates for NDI‐model, we hypothesize that the absence of microporosity restricts ion accessibility, leading to the more pronounced hysteresis. Conversely, NDI‐tryp exhibits a predominantly diffusion‐controlled mechanism, with *C*_o_ = 35% (Figures 5B and S23). This diffusion‐controlled charge‐storage mechanism aligns with the merging of peaks and strong hysteresis at high scan rates, indicating slower ion transport processes that require diffusion through the polymer, rather than redox states being accessible at interfaces. The Trasatti analysis therefore indicates that, although both NDI‐tryp and NDI‐spiro contain contorted units and high surface areas, the overall microstructure of the polymer dictates the material's accessible surface area, which in turn affects how efficiently redox‐active sites can contribute to energy storage. This interpretation is consistent with scanning electron micrographs, which show that NDI‐tryp has larger particle sizes and more agglomeration than NDI‐spiro and NDI‐model (Figures S31–S34). Thus, despite the similar microporous surface areas of NDI‐spiro and NDI‐tryp, the larger NDI‐tryp particles decrease the surface‐based redox processes, highlighting that a combination of microporosity and small particle size may be ideal for fast cycling.

Nyquist plots of EIS data further highlight differences in behavior between the polymers, with different magnitudes and frequencies of the impedance responses across the three materials. Complementary DRT analysis of EIS data provides additional insight by resolving the characteristic timescales that contribute to the impedance response. The DRT analysis used in this work was computed using a Gaussian radial basis function regularization model [41, 43]. Before cycling, NDI‐spiro half‐cells show the highest impedance, with a larger semicircle and pronounced low‐frequency tail in Nyquist plots (Figure 5D), indicative of substantial charge‐transfer resistance and slow mass transport [44, 45]. The DRT plot for NDI‐spiro under the same conditions shows that the material before cycling is dominated by a strong low‐frequency peak at long relaxation times (*τ* = 1 to 10 s), consistent with diffusion‐limited ion transport (Figure S21A). After 10 cycles at C/10, EIS shows a marked reduction in charge‐transfer resistance for NDI‐spiro, reflected by the contraction of the semicircle, consistent with improved charge‐transfer kinetics and enhanced electrolyte access to redox sites. The DRT plot of NDI‐spiro after cycling (Figure S21A) reveals a shift of the frequency peaks toward shorter timescales, associated with Ohmic resistance (*τ* = 0 s to 1 × 10^−2^ s) and with charge‐transfer and solid electrolyte interphase resistance (*τ* = 10 × 10^−2^ s to 1 s) [44, 45].

The Nyquist plot for NDI‐model also shows that the charge‐transfer resistance after cycling is reduced compared with the resistance before cycling (Figure 5F). However, DRT for NDI‐model reveals a persistent low‐frequency peak before and after cycling (Figure S21D), confirming that diffusion remains limiting despite the presence of a surface‐controlled mechanism overall. The resulting diffusion limitations account for the strong polarization observed at high scan rates in CV and support the role of microporosity in the other polymers in facilitating ion transport through surface‐based mechanisms. EIS data from NDI‐tryp shows behavior different from NDI‐spiro, with a charge‐transfer resistance that increases after 10 cycles (Figure 5E). DRT analysis for NDI‐tryp initially exhibits a more distributed response with reduced diffusion bottlenecks, but cycling induces the growth of low‐frequency contributions, suggesting the emergence of slower relaxation processes (Figure S21E). Overall, these results demonstrate that polymer topology critically governs both initial transport behavior and its evolution during cycling, with spiro‐linked architecture enabling the most effective ion accessibility at both slow and fast cycling rates.

### 2.4 Effect of Redox‐Active Unit on Electrochemical Behavior

**FIGURE 6:**
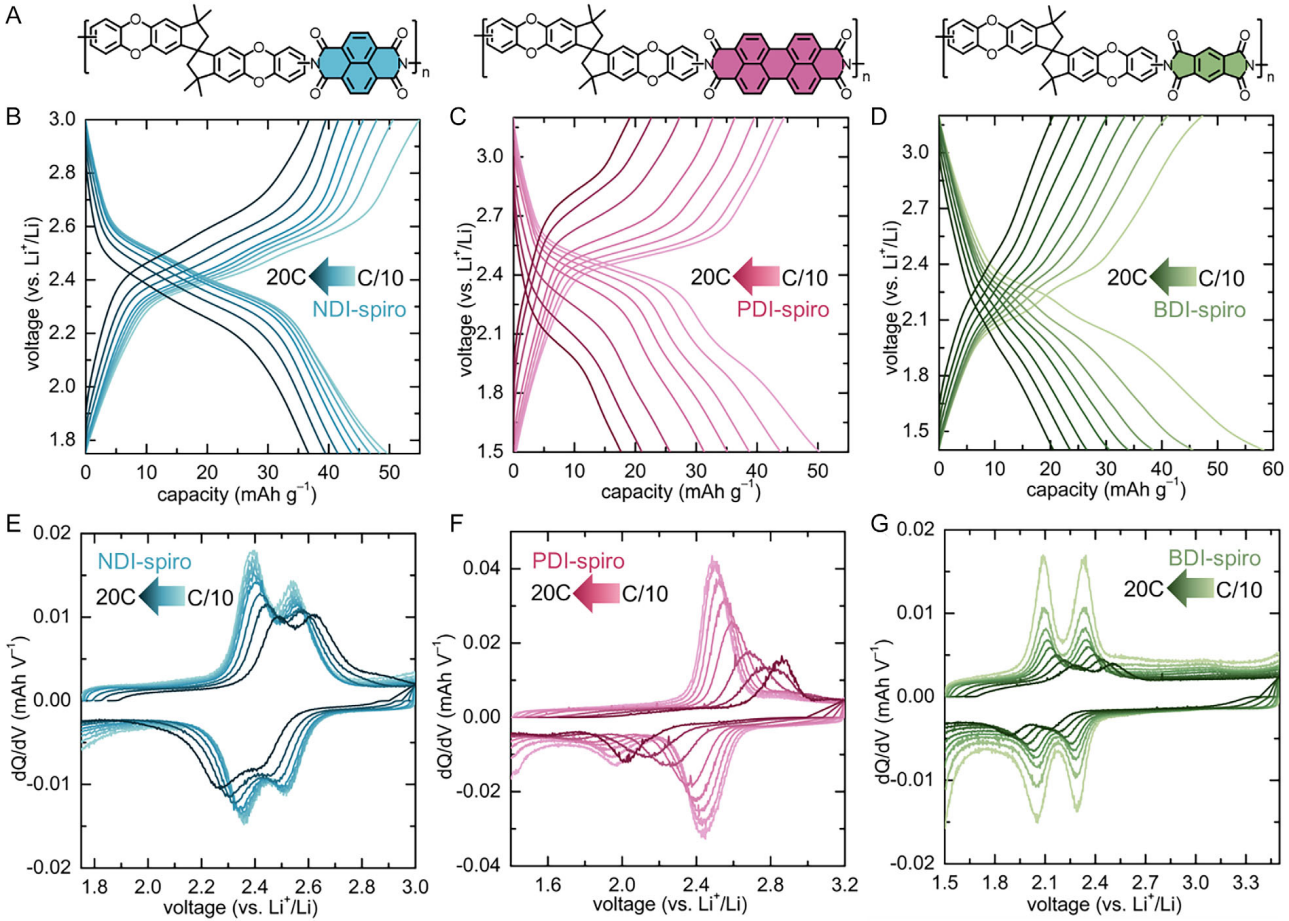
(A) Structures of NDI‐spiro, PDI‐spiro, and BDI‐spiro. Galvanostatic charge–discharge profiles at different cycling rates for (B) NDI‐spiro, (C) PDI‐spiro, and (D) BDI‐spiro and differential capacity plots at different cycling rates of (E) NDI‐spiro, (F) PDI‐spiro, and (G) BDI‐spiro.

Based on the high rate capability and low polarization of NDI‐spiro, we also evaluate the impact of different redox active units on battery cycling behavior in half cells, in which Li metal serves as both reference and counter electrode (Figure 6A). The spiro‐based polymers were synthesized via condensation polymerization of a redox‐active anhydride naphthalenetetracarboxylic dianhydride (NTCDA), perylenetetracarboxylic dianhydride (PTCDA), or pyromellitic dianhydride (PMDA) with the amine‐functionalized‐spiro linker (Figure 2C). The resulting polymers (NDI‐spiro, PDI‐spiro, and BDI‐spiro) had surface areas of 273, 190, and 75 m^2^ g^−1^, respectively (Figure 2B).

As in the variable linker series, we assessed cycling and rate capabilities in the redox‐active unit series using GCPL at various rates. After 10 cycles at C/10, all three spiro‐based polymers exhibit well‐defined voltage features in the GCPL profiles, indicating charge storage by the redox‐active species under conditions where diffusion is not limiting (Figure 6). At less kinetically demanding conditions (i.e., slow rates), the three materials perform similarly in terms of achieved capacities relative to their theoretical capacities. PDI‐spiro achieves 79% of its theoretical capacity (48.4 vs. 61.3 mAh g^−1^) (Figures 6C and S19A), and BDI‐spiro reaches 76% of its theoretical capacity with a capacity (58.3 vs. 76.4 mAh g^−1^) (Figures 6D and S20A). These values are similar to those of NDI‐spiro under the same conditions (71% of its theoretical capacity) (Figures 6B and S16A). As cycling rate increases from C/10 to 20C, NDI‐spiro maintains the most well‐defined features (Figure 6B), whereas PDI‐spiro and BDI‐spiro exhibit a more pronounced loss of feature definition (Figure 6C,D). The loss of feature definition reveals kinetic limitations at fast rates that lead to low capacities, reflected in the low capacity achieved with increasing rate.

These trends across the series are consistent in differential capacity (dQ/dV) analysis. During slow cycling (C/10), BDI‐spiro displays two discharge features, at 2.05 and 2.29 V, and two charge features, at 2.10 and 2.33 V (Figure S20B). PDI‐spiro exhibits a dominant discharge feature at 1.97 and 2.44 V and one feature during charge, centered at 2.49 V (Figure S19B). NDI‐spiro shows the most symmetric behavior between discharge and charge, with closely spaced discharge features at 2.35 and 2.51 V and charge features at 2.40 and 2.54 V (Figure S16B). As the cycling rate increases to 20C, NDI‐spiro largely preserves its voltage features, shifting modestly to lower discharge voltages (2.27 and 2.41 V) and higher charge voltages (2.48 and 2.62 V), indicative of a modest increase in hysteresis and, thus, relatively faster kinetics (Figure 6E). In contrast, PDI‐spiro exhibits the most pronounced polarization, with discharge features shifting to 1.59 and 2.04 V and the charge feature shifting to 2.86 V, reflecting severe kinetic limitations at fast rates (Figure 6F). BDI‐spiro shows intermediate behavior, with discharge features at 1.89 and 2.11 V and charge features at 2.27 and 2.48 V, retaining two redox processes more clearly than PDI‐spiro, but with lower overall utilization of redox‐active sites, as reflected in its reduced capacity (Figure 6G).

**FIGURE 7:**
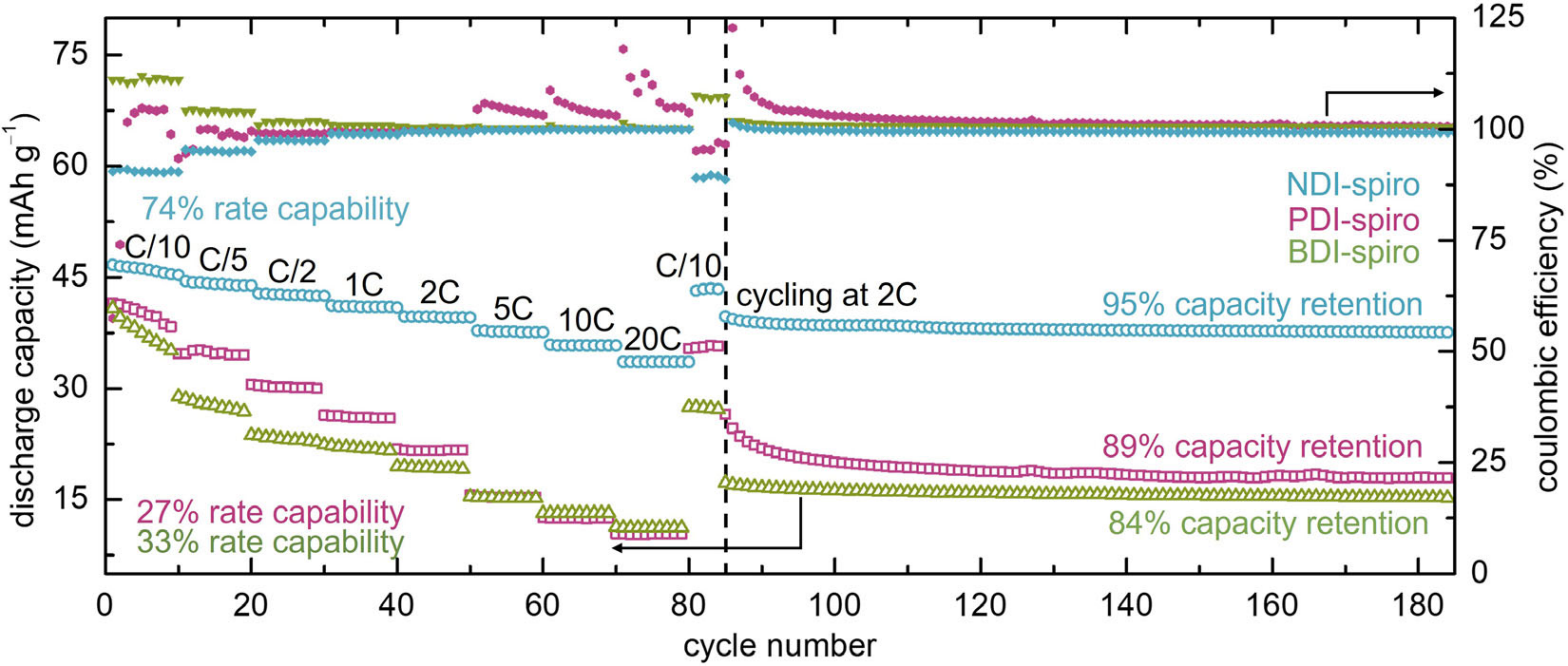
Average discharge capacities (bottom left) and Coulombic efficiencies (top left) of NDI‐spiro, PDI‐spiro, and BDI‐spiro during variable rate cycling (from C/10 to 20C). Average discharge capacities (bottom right) and Coulombic efficiencies (top right) during cycling at 2C for an additional 100 cycles. The capacities and Coulombic efficiency values at each cycle number are the average of three representative cells for each material (plotted with standard deviations in Figure S28).

The qualitative observations about kinetic limitations seen with differential capacity analysis are also evident in the materials’ rate capabilities. From C/10 to 20C, PDI‐spiro and BDI‐spiro exhibit poor rate capabilities, 27% and 33%, respectively (Figure 7, left), which are significantly lower than NDI‐spiro (74%). This superior rate capability correlates with the higher internal surface area of NDI‐spiro (273 m^2^ g^−1^) (Figure 1C), which provides more effective microporous pathways for Li^+^ transport and minimizes diffusion limitations at fast cycling rates. In contrast, PDI‐spiro and BDI‐spiro lose capacity with increasing rate, which we attribute to their lower surface areas that, in turn, decrease ion accessibility.

To assess capacity retention of the materials through extended cycling, batteries for each polymer were evaluated over 100 additional cycles at a 2C rate. NDI‐spiro retains 95% of its initial capacity, PDI‐spiro retains 89%, and BDI‐spiro retains 84% (Figure 7, right). These capacity retention values show that the materials remain stable over extended cycling, indicating minimal degradation or dissolution in the electrolyte [14, 38]. Under constant rate cycling at 2C, PDI‐spiro achieves 57% of its theoretical capacity, exceeding that of NDI‐spiro (53%) and BDI‐spiro (21%) (Figure 7, right). The higher achieved percent of theoretical capacity for PDI‐spiro compared to NDI‐spiro may result from its higher electronic conductivity, a known consequence of differences in the polymer backbones [24]. Conjugation increases along an extended aromatic system, such as PDI, which has been associated with higher electronic conductivity in previous studies of PDI‐based networks in electrochromic devices. From previous reports, the redox‐active units we consider here are expected to follow the order of electronic conductivity: PDI > NDI > BDI. The extended π‐conjugation of the PDI unit enhances electronic delocalization and conductivity relative to NDI and BDI, enabling more efficient charge transport during sustained cycling at a fixed, moderately fast rate. Consequently, PDI‐spiro achieves higher redox utilization despite its lower microporosity. BDI‐spiro, which combines limited conjugation with a lower accessible surface area, remains kinetically disadvantaged in both slow and fast regimes. Overall, these data show that intrinsic microporosity and backbone conjugation govern complementary aspects of high‐rate performance. While electronic conductivity from conjugation benefits capacity at slow and moderate rates, the combination of microporosity and high surface area is important for fast rate capabilities.

Since the linker structure is identical across the spiro‐based polymers, a comparison of the Coulombic efficiencies of NDI‐spiro, PDI‐spiro, and BDI‐spiro highlights the influence of the redox active unit on electrochemical reversibility. NDI‐spiro, PDI‐spiro, and BDI‐spiro exhibit high Coulombic efficiencies during variable rate cycling, with average Coulombic efficiencies of 97% for NDI‐spiro, 101% for PDI‐spiro, and 103% for BDI‐spiro (Figure 7, left). During the cycling stability experiment, NDI‐spiro has a Coulombic efficiency of 99%, PDI‐spiro shows 102%, and BDI‐spiro shows 101% (Figure 7, right). These high values are comparable with that of NDI‐spiro, indicating highly reversible electrochemical process. Coulombic efficiencies slightly exceeding 100% have occasionally been reported for organic electrode materials and are generally attributed to minor experimental uncertainties or kinetic effects associated with charge–discharge cutoff conditions rather than a fundamental increase in charge storage [45].

### 2.5 Ion‐Transport Mechanisms in the Redox‐Active Unit Series

**FIGURE 8:**
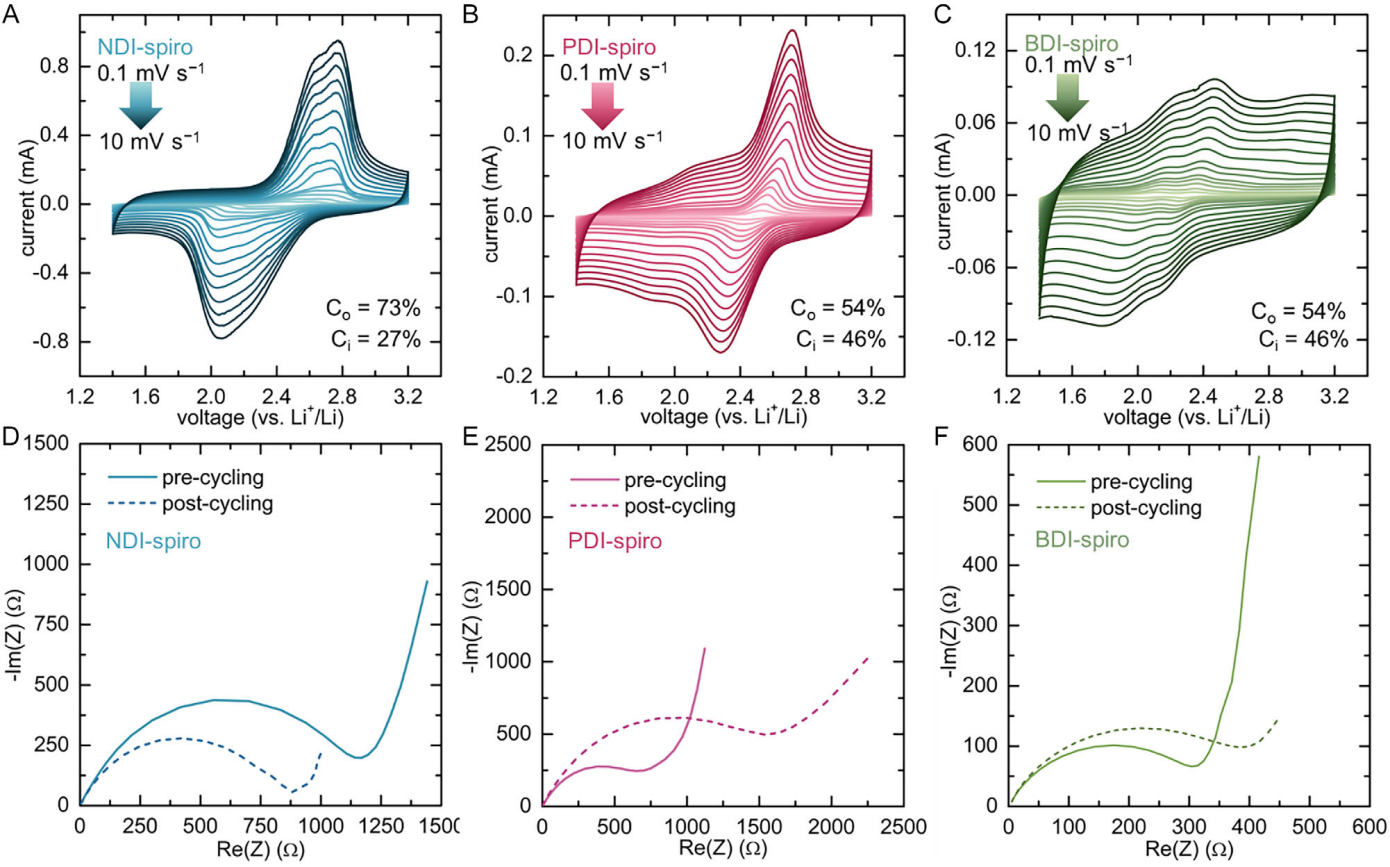
Cyclic voltammetry plots from 0.1 to 10 mV s^−1^ for (A) NDI‐spiro, (B) PDI‐spiro, and (C) BDI‐spiro. Nyquist plots of precycling (solid lines) and postcycling (dotted lines) of (D) NDI‐spiro, (E) PDI‐spiro, and (F) BDI‐spiro. The CVs were taken at 0.10, 0.25, 0.50, 0.75, 1.00, 1.5, 2.00, 3.00, 4.00, 5.00, 6.00, 7.00, 8.00, 9.00, and 10 mV s^−1^.

To further study the kinetic limitations and charge‐storage mechanisms that impact the rate capability of the materials, we used CV, EIS, and DRT. The CV responses of the three spiro‐linked polymers reveal differences in current magnitude and redox definition (Figure 8A–C). Under the same experimental conditions as NDI‐spiro, PDI‐spiro exhibits one current peak at slow scan rates (0.1 mV s^−1^) with a hysteresis of 0.10 V. With a faster scan rate of 10 mV s^−1^, the hysteresis also increases to 0.44 V. BDI‐spiro shows two sharp peaks at slow scan rates with a hysteresis of 0.09 and 0.08 V, which increases to 0.41 and 0.33 V with a scan rate of 10 mV s^−1^ (Table S3). These data suggest that all three materials show similar electrochemical activity at slow scan rates (0.1 mV s^−1^). However, when increasing the scan rate, NDI‐spiro shows the lowest hysteresis, indicating better redox accessibility than PDI‐spiro and BDI‐spiro.

Trasatti analysis was used to assess the charge‐storage mechanisms of each material, which reveals differences in the storage mechanism depending on the redox‐active unit (Table S2). NDI‐spiro has 73% surface‐controlled (*C*_o_) and 27% diffusion‐controlled (*C*_i_) (Figures 8A and S22). Despite having the same contorted linker, PDI‐spiro and BDI‐spiro exhibit lower surface contributions, 54.4% and 54.0%, respectively (Figures 8B,C, S25, and S26). NDI‐spiro has the highest surface area compared to PDI‐spiro and BDI‐spiro (Figure 2C), which may allow a greater contribution from surface‐controlled processes and, therefore, less impact from diffusion limitations, leading to better rate capability.

The EIS and DRT data before and after cycling further confirm the observed differences in charge‐storage mechanism with redox‐active unit. PDI‐spiro and BDI‐spiro Nyquist plots show smaller charge‐transfer resistance before cycling than in NDI‐spiro (Figure 8D–F). Analysis of DRT data shows that both PDI‐spiro and BDI‐spiro exhibit a strong low‐frequency peak at long relaxation times: Although the intensities of these peaks decrease after several cycles, the dominant low‐frequency peak remains (Figure S21B,C). These results indicate that slow processes dominate the impedance response after cycling for our PDI‐ and BDI‐based polymers, consistent with their diffusion‐controlled charge‐storage mechanism from Trasatti analysis.

## 3 Conclusions

We find that fast cycling capabilities in polymer electrodes benefit from high surface areas, which promote surface‐controlled redox mechanisms and increase accessible redox states. We show an NDI‐based PIM (NDI‐spiro) with high surface area (associated with microporosity) and small particle size has a surface‐dominated redox mechanism, which results in a sustained capacity and energy‐efficient redox features from slow to fast cycling rates, showing 74% of the C/10 capacity is retained at 20C. In contrast, polymer systems with low microporosity (PDI‐spiro, BDI‐spiro) or larger particles (NDI‐tryp) have dominant diffusion‐controlled redox mechanisms and poor rate capabilities (e.g., only 33% of the slow‐rate capacity retained at fast rates for BDI‐spiro). A model system (NDI‐model) with a noncontorted linker lacks microporosity and has low surface area, resulting in low capacity, even at slow cycling rates. When we consider another contorted linker, NDI‐tryp, we find that despite its high initial capacity at slow rates (77% of its theoretical capacity at C/10), it cannot retain this capacity at higher rates. This poor rate capability is a result of NDI‐tryp's diffusion‐controlled charge‐storage mechanism, despite its high surface area, which we attribute to its large particle size and high degree of particle agglomeration. We observe similar behavior in other spiro‐based polymers with PDI and BDI redox units. Specifically, they also show good capacity at slow rates, but inferior rate capabilities compared to NDI‐spiro. While the contorted spiro linker consistently introduces microporosity that allows efficient utilization of redox sites at slow rates, as the cycling rate increases, only the NDI‐spiro PIM provides a surface‐controlled charge‐storage mechanism that enables accessible capacity at fast cycling conditions. Together, this work establishes that optimizing both intrinsic microporosity and redox core chemistry is essential for designing high‐performance organic battery electrodes.

## Author Contributions

**Eva Vásquez‐Barillas** conceived the experiments, synthesized and characterized the monomers and polymers, assembled cells, designed and executed battery performance experiments, and analyzed battery data. **Ani N. Davis** assisted with electrode formulation and battery assembly. **Kiana A. Treaster** and **Swapnanil Goswami** assisted with material characterization. **A M Mahmudul Hasan** assisted with the synthesis of polymers. **Amrit Kaur** assisted with the synthesis of monomers. **Gabriel Carson** assisted in battery electrode processing. **Kausturi Parui** and **John D. Langhout** assisted with battery analysis. **Joshua D. Marquez** and **Rachel H. Bianculli** assisted with monomer characterization. **Eva Vásquez‐Barillas** wrote the initial drafts of the manuscripts. **Megan M. Butala** and **Austin M. Evans** contributed to experimental design and supervised this work. All authors contributed to the writing of this manuscript.

## Funding

This study was supported by Army Research Office (W911NF2410186 and W911NF2410186) and US Department of Defense (W911NF2310260).

## Conflicts of Interest

The authors declare no conflicts of interest.

## Supporting information

Supplementary Material

## Data Availability

The data supporting this article have been included as part of the Supporting Information, and the data that support the findings of this study are available from the corresponding authors upon reasonable request.
