# Supplementary material for: Polymers of Intrinsic Microporosity as Organic Cathodes for Fast‐Charging Lithium‐Ion Batteries
Source: Small Sci. 2026 Aug 13;6(8):e70364. doi: 10.1002/smsc.70364 (PMC13489316; doi:10.1002/smsc.70364)
Supplement: Supplementary file 1 [file SMSC-6-e70364-s001.pdf]

Supporting Information

Polymers of Intrinsic Microporosity as Organic Cathodes for Fast-charging Lithium-ion Batteries

Eva Vásquez-Barillas, Ani N. Davis, A M Mahmudul Hasan, Amrit Kaur, Kiana A. Treaster, Kausturi Parui, John D. Langhout, Rachel H. Bianculli, Joshua D. Marquez, Gabriel Carson, Austin M. Evans*, Megan M. Butala*

Eva Vásquez-Barillas, Ani N. Davis, A M Mahmudul Hasan, Amrit Kaur, Kiana A. Treaster, Rachel H. Bianculli, Joshua D. Marquez,Austin M. Evans*

George and Josephine Butler Polymer Laboratory, Department of Chemistry, University of Florida, Gainesville, FL 32611, USA

austinevans@chem.ufl.edu

Kausturi Parui, John D. Langhout, Gabriel Carson, Megan M. Butala*

Department of Materials Science and Engineering, University of Florida, Gainesville, FL 32611, USA

mbutala@ufl.edu

Table of Contents

Materials and Instrumentation **5**

Instrumentation **5**

Nuclear Magnetic Resonance (NMR) Spectroscopy. **5**

Fourier Transform Infrared (FTIR) Spectroscopy. **5**

Electrochemical Analysis. **5**

CO_2 Sorption Isotherms. **5**

Mass Spectrometry (MS). .... Error! Bookmark not defined.

Monomer Synthesis **6**

Synthesis of SBIDN **6**

Synthesis of SBIDA **7**

Synthesis of naphthalopleiadene (NP) **8**

Synthesis of NPDN **9**

Synthesis of NPDA **10**

Polymer Synthesis **11**

NDI-spiro synthesis **11**

PDI-spiro synthesis **12**

BDI-spiro synthesis **13**

Synthesis of naphthopleiadene based polymer of intrinsic microporosity (NDI-tryp) **14**

Model Compound Synthesis **15**

Synthesis of NDI-model **15**

Polymer Characterization **16**

Electrode Preparation **21**

Coin Cell Assembly **21**

Electrochemical Measurements **21**

Galvanostatic cycling with potential limitations **21**

Cyclic voltammetry **21**

Electrochemical impedance spectroscopy **22**

Supporting Figures **23**

Nuclear Magnetic Resonance Spectroscopy **37**

Matrix-Assisted Laser Desorption/Ionization .... Error! Bookmark not defined.

References **43**

List of Supporting Figures

Figure S1. Scheme of the synthesis of SBIDN. 6
Figure S2. Scheme for the synthesis of SBIDA. 7
Figure S3. Scheme of the synthesis of naphtalopleiadene (NP). 8
Figure S4. Scheme of the synthesis of NPDN. 9
Figure S5. Scheme of the synthesis of NPDA. 10
Figure S6. Scheme of the synthesis of NDI-spiro. 11
Figure S7. Scheme of synthesis of PDI-spiro. 12
Figure S8. Scheme of synthesis of BDI-spiro. 13
Figure S9. Scheme of synthesis of NDI-tryp. 14
Figure S10. Scheme of synthesis of NDI-model. 15
Figure S11. FTIR of NDI-spiro polymer of intrinsic microporosity, starting materials (SBIDA and NTCDA), and precursor (SBIDN). 16
Figure S12. FTIR of PDI-spiro polymer of intrinsic microporosity, starting materials (SBIDA and PTCDA), and precursor (SBIDN). 17
Figure S13. FTIR of BDI-spiro polymer of intrinsic microporosity, starting materials (SBIDA and PMDA), and precursor (SBIDN). 18
Figure S14. FTIR of NDI-tryp polymer of intrinsic microporosity, starting materials (NPDA and NTCDA), and precursors (NPDN and NPOH). 19
Figure S15. FTIR spectra of NDI-model polymer of intrinsic microporosity and starting materials (BPDA and NTCDA). 20
Figure S16. A. Galvanostatic cycling with potential limitations and **B.** differential capacity plot of NDI-spiro at C/10 and 20C. 23
Figure S17. A. Galvanostatic cycling with potential limitations and **B.** differential capacity plot of NDI-tryp at C/10 and 20C. 24
Figure S18. A. Galvanostatic cycling with potential limitations and **B.** differential capacity plot of NDI-model at C/10 and 20C. 25
Figure S19. A. Galvanostatic cycling with potential limitations and **B.** differential capacity plot of PDI-spiro at C/10 and 20C. 26
Figure S20. A. Galvanostatic cycling with potential limitations and **B.** differential capacity plot of BDI-spiro at C/10 and 20C. 27
Figure S21. Distribution of relaxation times (DRT) plots for **A.** NDI-spiro, **B.** PDI-spiro, **C.** BDI-spiro, **D.** NDI-model, and **E.** NDI-tryp. 28
Figure S22. Linear regression used for the Trasatti analysis of NDI-spiro. **A.** Total capacitance and **B.** outer capacitance contribution. 29
Figure S23. Linear regression used for the Trasatti analysis of NDI-tryp. **A.** Total capacitance and **B.** outer capacitance contribution. 30
Figure S24. Linear regression used for the Trasatti analysis of NDI-model. **A.** Total capacitance and **B.** outer capacitance contribution. 31
Figure S25. Linear regression used for the Trasatti analysis of PDI-spiro. **A.** Total capacitance and **B.** outer capacitance contribution. 32
Figure S26. Linear regression used for the Trasatti analysis of BDI-spiro. **A.** Total capacitance and **B.** outer capacitance contribution. 33
Figure S27. Average discharge capacities (left) during variable rate cycling (from C/10 to 20C) and (right) during cycling at 2C for an additional 100 cycles for NDI-spiro, NDI-tryp and NDI-model. 34
Figure S28. Average discharge capacities (left) during variable rate cycling (from C/10 to 20C) and (right) during cycling at 2C for an additional 100 cycles for NDI-spiro, PDI-spiro, and BDI-spiro. 35
Figure S29. ^{1}H NMR (400 MHz, d-DMSO) of SBIDN. 37
Figure S30. ^{1}H NMR (400 MHz, d-DMSO) of SBIDA. 38
Figure S31. ^{1}H NMR (400 MHz, d-DMSO) of NP. 39
Figure S32. ^{1}H NMR (400 MHz, $CDCl_3$) of NPDN. 40
Figure S33. ^{13}C NMR (100 MHz, $CDCl_3$) of NPDN. 41

Figure S34. ^{1}H NMR (400 MHz, $CDCl_3$) of NPDA. 42

Table S1. Data for coin cell batteries used in electrochemical measurements. 36
Table S2. Total, outer, and inner capacitance obtained by Trasatti analysis. 36
Table S3. Oxidation and reduction potentials (vs. Li^+/Li) with hysteresis from differential capacity plots....... 36

Materials and Instrumentation

All materials were purchased from Fisher Scientific, Sigma Aldrich, Ambeed, TCI Chemicals, or MSE Supplies. Deuterated solvents were purchased from Cambridge Isotope Laboratories. All chemicals were used as purchased unless otherwise noted. Reactions were performed under an inert reaction atmosphere in flame-dried round-bottom flasks unless otherwise specified.

Instrumentation

Nuclear Magnetic Resonance (NMR) Spectroscopy. ^{1}H NMR spectra were acquired on a Bruker 400 MHz spectrometer and recorded at 25 °C. All chemical shifts were calibrated using residual protic solvent as internal reference (DMSO-d_6: 2.54 ppm for ^{1}H NMR and 39.7 ppm for ^{13}C NMR. Methanol-d_4: 3.31 ppm for ^{1}H NMR and 49.31 ppm for ^{13}C NMR).

Fourier-Transform Infrared (FTIR) Spectroscopy. FTIR spectra were collected with an Agilent Cary 630 FT-IR spectrometer equipped with a diamond ATR attachment. The spectra of samples were recorded in a wavenumber range of 4000-600 cm^{-1} with a resolution of 4 cm^{-1}. The presented spectra have undergone background subtraction.

Electrochemical Analysis. Batteries were cycled on a Bio-Logic Variable Multichannel Potentiostat VMP3 under galvanostatic conditions, as well as cyclic voltammetry. The same potentiostat was used to perform electrochemical impedance spectroscopy (EIS).

CO_2 Sorption Isotherms. Sorption isotherms were collected on a Micromeritics 3Flex High Performance Gas Analyzer. Approximately 30–50 mg of polymer powder was transferred to a pre-weighed, dried analysis tube and sealed with a check seal cap connected to a glass filler rod. The tube was weighed again, and the sample was activated by evacuation at 105 °C for 24 h until the outgas rate was ≤ 0.3 μm Hg min^{-1}. The tube was then reweighed to determine the mass of the activated sample and subsequently transferred to the analysis port. The sorption measurements were carried out in the presence of CO_2 gas. CO_2 isotherms were generated by incremental exposure to carbon dioxide up to 760 mmHg (1 atm) in a dry ice/acetone (198 K) bath. Brunauer-Emmett- Teller (BET) surface areas were calculated from the linear region of the CO_2 isotherm.

Monomer Synthesis

Synthesis of SBIDN

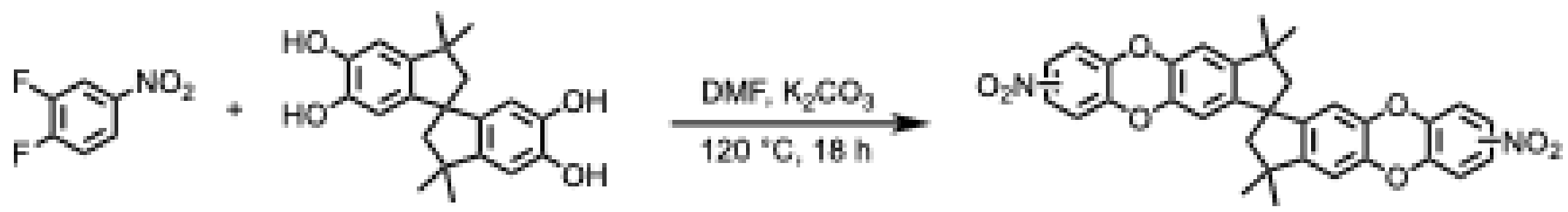

Figure S1. Scheme of the synthesis of SBIDN.

This procedure was adapted from a previous report.[11] Commercially available 5,5',6,6'-tetrahydroxy-3,3,3',3'-tetramethyl–1,1'-spirobisindane (1.50 g, 4.41 mmol, 1.00 equiv) and 3,4-difluronitrobenzene (1.40 g, 8.80 mmol, 2.00 equiv) were charged in a 100 mL flame-dried double-neck round-bottom flask under a N_2 atmosphere. Dry dimethyl-formamide (DMF, 20 mL) and potassium carbonate (K_2CO_3, 5.17 g, 37.4 mmol, 8.50 equiv) were added to the round-bottom flask. The mixture was stirred for 2 h at room temperature (22 °C), then the temperature was increased to 80 °C and maintained for 30 min. After that, the temperature was increased by 20 °C every 30 min until the reaction mixture reached 120 °C, and it was kept at 120 °C for 18 h. A yellow and turbid reaction mixture emerged from these conditions. The reaction mixture was removed from the heat and allowed to cool to 22 °C. The product was isolated by precipitation from 150 mL of deionized water, then gravity-filtered through filter paper in a plastic funnel and washed 3 times with 20 mL of deionized water. The isolated product was then recrystallized in hot methanol (50 °C, 30 mL). A yellow powder was then collected by gravity filtration through filter paper in a plastic funnel and dried in the vacuum oven (60 °C, 24 h) to yield 1.94 g of the target compound (76.1 % yield). ^{1}H NMR data was consistent with the previous report[2].

^{1}H NMR (400 MHz, *d*-DMSO) δ 7.87 (ddd, J = 9.0, 6.5, 2.7 Hz, 2H), 7.77 (d, J = 2.7 Hz, 1H), 7.68 (d, J = 2.6 Hz, 1H), 7.19 (d, J = 9.0 Hz, 1H), 7.10 (d, J = 8.9 Hz, 1H), 6.97 (d, J = 11.2 Hz, 2H), 6.38 (d, J = 13.8 Hz, 2H), 2.29 (d, J = 13.1 Hz, 2H), 2.14 (d, J = 13.1 Hz, 2H), 1.35 (s, 6H), 1.28 (s, 6H).

FTIR (ATR) 2954, 2951, 1673, 1623, 1587, 1525, 1491, 1426, 1316, 1270, 1075 cm^{-1}.

Synthesis of SBIDA

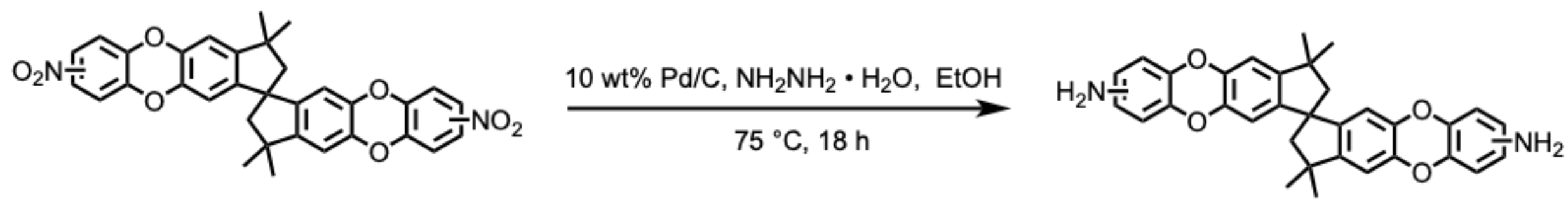

Figure S2. Scheme for the synthesis of SBIDA.

This procedure was adapted from a previous report.[1]

SBIDN (1.91 g, 3.30 mmol, 1 equiv), and 10% w/w palladium on carbon (Pd/C, 0.19 g) were charged in a 250 mL flame-dried round-bottom flask in a N_2 atmosphere, then ethanol was added (EtOH, 95 mL). After, hydrazine monohydrate (2 mL, 85.9 mmol, 26 equiv) was added dropwise at room temperature (22 °C), then the temperature was increased to 60 °C and maintained for 15 min. Following this step, the reaction mixture was stirred at 75 °C for 18 h. The reaction was removed from the heat and allowed to cool to room temperature (22 °C), and Pd/C was removed by filtering through a celite pad and the pad was washed once with 20 mL of ethanol. The filtrate, which contains the crude product, was concentrated by rotary evaporation until approximately 5 mL of solvent remained, then diluted to 100 mL with deionized water, yielding a white precipitate. The product was collected by gravity filtration through filter paper in a plastic funnel, washed 3 times with 20 mL of deionized water, and then dried in the vacuum oven (60 °C, 24 h) to yield 1.14 g of the target compound (66.5% yield). ^{1}H NMR data was consistent with the previous report.[2]

^{1}H NMR (400 MHz, DMSO) δ 6.83 (d, J = 1.3 Hz, 1H), 6.78 (d, J = 1.2 Hz, 1H), 6.57 (dd, J = 8.5, 1.1 Hz, 1H), 6.26 (d, J = 1.5 Hz, 1H), 6.21 (d, J = 1.4 Hz, 1H), 6.19 – 6.10 (m, 4H), 4.96 (d, J = 5.4 Hz, 4H), 2.25 (d, J = 13.1 Hz, 2H), 2.10 (d, J = 13.0 Hz, 2H), 1.33 (t, J = 2.1 Hz, 5H), 1.26 (s, 6H).

FTIR (ATR) 2953, 2862, 1675, 1587, 1524, 1485, 1320 cm^{-1}.

Synthesis of naphthalopleiadene (NP)

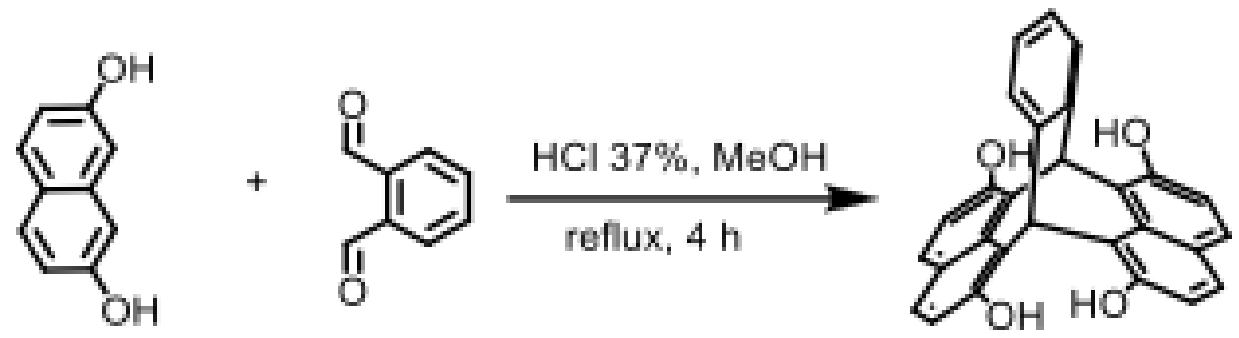

Figure S3. Scheme of the synthesis of naphtalopleiadene (NP).

This procedure was adapted from a previous report.[3] Commercially available naphthalene-2,7-diol (1.18 g, 7.37 mmol, 2 equiv) and phtalaldehyde (0.49 g, 3.68 mmol, 1 equiv) were charged in a 50 mL flame dried two-neck round bottom flask equipped with a reflux condenser in a N_2 atmosphere. Methanol (MeOH, 15 mL) was added, and the reaction was stirred at room temperature (22 °C) for 10 min, concentrated chlorohydric acid (HCl 12 M, 0.1 mL) was added dropwise to the reaction mixture and the solution changed color from pale-yellow to a transparent solution. Following that, the temperature was increased to 85 °C and stirred for 4 h. During this time, the reaction mixture changed color to a deep red. After the reaction was complete, a precipitate formed. The reaction mixture was removed from the heat and allowed to cool to room temperature (22 °C), then put into an ice bath to cool to 0 °C. The precipitate was collected by gravity filtration and washed 3 times with cold ethanol to obtain a white powder. The powder was then dried in the vacuum oven (60 °C, 24 h), to yield 0.62 g of the desired product (40.3% yield). ^{1}H NMR data was consistent with the previous report.[3]

^{1}H NMR (400 MHz, DMSO) δ 9.95 (s, 4H), 7.46 (d, *J* = 8.7 Hz, 4H), 7.37 (dd, *J* = 5.5, 3.4 Hz, 2H), 7.15 (dd, *J* = 5.5, 3.3 Hz, 2H), 6.95 (d, *J* = 8.7 Hz, 4H), 6.80 (s, 2H).

FTIR (ATR) 3159, 1614, 1511, 1441, 1335, 1311, 1264, 1187, 1146, 1113, 1032 cm^{-1}.

Synthesis of NPDN

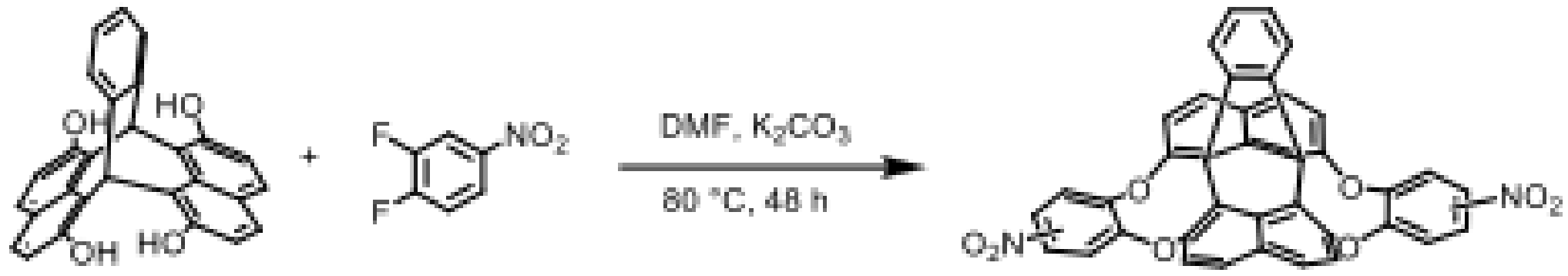

Figure S4. Scheme of the synthesis of NPDN.

This procedure was adapted from a previous report.[3] NP (1.00 g, 2.39 mmol, 1 equiv) and difluoronitrobenzene (0.53 mL, 4.78 mmol, 2 equiv) were charged in aflame-dried double-neck round bottom flask under N_2 atmosphere. Dry dimethyl-formamide (DMF, 40 mL) and potassium carbonate (K_2CO_3, 2.70 g) were added to the reaction mixture. The mixture was stirred for 2 h at room temperature (22 °C). Then the temperature was increased by increments of 40 °C every 30 min until the temperature reached 80 °C and the reaction mixture was kept at 80 °C for 48 h. A yellow reaction mixture was formed and the product was isolated by precipitation into 100 mL of deionized water yielding a light-yellow solid. The solid was isolated by gravity filtration, washed 2 times with 20 mL of deionized water, then washed with 20 mL of methanol, and dried in the vacuum oven (60 °C, 24 h) to yield 1.17 g of the desired product (74.7 % yield).

^{1}H NMR (400 MHz, $CDCl_3$) δ 8.11 – 8.03 (m, 3H), 7.79 – 7.73 (m, 3H), 7.66 (d, *J* = 8.9 Hz, 3H), 6.97 – 6.89 (m, 6H), 6.79 (dd, *J* = 5.5, 3.4 Hz, 2H), 6.54 – 6.48 (m, 3H).

^{13}C NMR (100 MHz, $CDCl_3$) δ 153.9 – 153.3 (3C), 150.6 (1C), 145.6 (1C), 143.1 (1C), 140.9 (1C), 140.4 (1C), 131.6 – 130. 5 (6C), 130.5 – 130.5 (4C), 130.0 (1C), 129.1 (1C), 128.8 – 128.0 (5C), 125.7 (1C), 122.2 – 121.8 (2C), 121.3 – 120.7 (4C), 120.7 – 120.1 (3C), 119.6 – 119.2 (2C), 119.1 (1C), 118.6 (1C), 114.0 (1C), 113.3 (1C).

FT-IR (ATR) 1600, 1531, 1492, 1432, 1352, 1327, 1265, 1225, 1223, 1133, 1114, 1076, 942 cm^{-1}.

Synthesis of NPDA

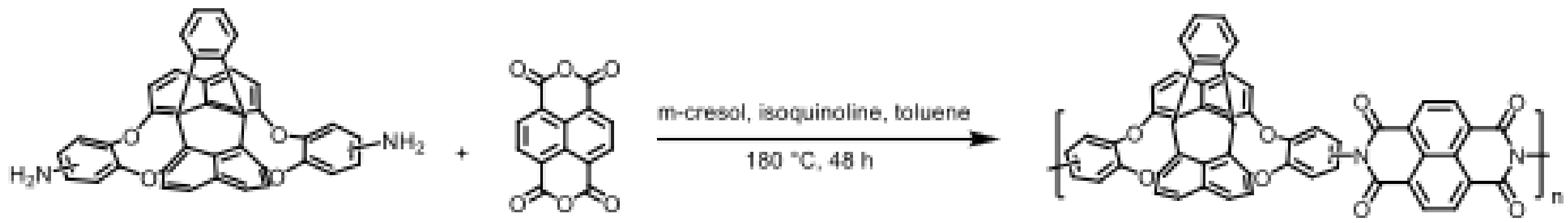

Figure S5. Scheme of the synthesis of NPDA.

This procedure was adapted from a previous report.[4] NPDN (0.20 g, 0.30 mmol, 1 equiv) and tetrahydrofuran (THF, 15 mL) were added to a flame-dried two-neck round-bottom flask equipped with a condenser, and the system was gassed-degassed 3 times. The reaction mixture was stirred at room temperature (22 °C) for 30 min, then the mixture was cooled in an ice bath to 0°C, and 5% w/w Raney nickel (2.5 mL) was added dropwise. The reaction was stirred for 15 min. After this time, hydrazine monohydrate ($NH_2NH_2 \cdot H_2O$, 1.3 mL, 4.26 mmol, 14 equiv) was dropwise added. After 30 min of stirring, the reaction mixture was removed from the ice bath, allowed to warm to room temperature (22 °C), and stirred for 1 hr. After, the temperature was increased to 75 °C and stirred for 24 hr. Following this, the reaction mixture was removed from the heat, allowed to cool to room temperature (22 °C), and the Raney nickel was removed by filtering through a celite pad. The solvent was evaporated from the filtrate under vacuum after which a sticky solid was obtained; 5 mL of hexane was added, and the mixture was filtered to yield a brown powder. 10 mL of chloroform was added to the powder to remove impurities, and the mixture was filtered again to obtain a beige powder. The powder was dried in a vacuum oven (60 °C, 24 h), yielding 0.08 g (44.0 %) of the desired product.

^{1}H NMR (400 MHz, $CDCl_3$) δ 7.49 (s, 1H), 7.39 (d, *J* = 9.0 Hz, 2H), 7.30 (dd, *J* = 5.5, 3.4 Hz, 1H), 7.05 (dd, *J* = 5.5, 3.2 Hz, 1H), 6.81 (d, *J* = 8.8 Hz, 2H), 6.57 (t, *J* = 8.7 Hz, 2H), 6.42 (dd, *J* = 11.8, 2.7 Hz, 2H), 6.27 – 6.18 (m, 2H), 3.53 (s, 4H).

FTIR (ATR) 3347, 1662, 1613, 1591, 1515, 1454, 1424, 1328, 1260, 1215, 1158, 1112, 1090, 955 cm^{-1}.

Polymer Synthesis
NDI-spiro synthesis

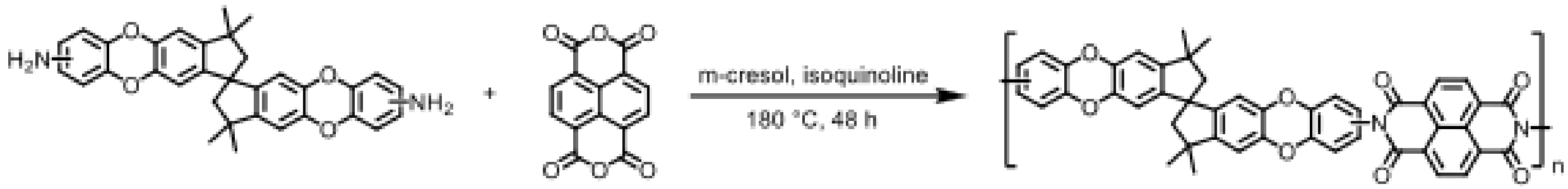

Figure S6. Scheme of the synthesis of NDI-spiro.

This procedure was adapted from a previous report.[2] SBIDA (0.60 g, 1.16 mmol, 1 equiv) and 1,4,5,8-naphthalenetetracarboxylic dianhydride (0.31 g, 1.16 mmol, 1 equiv) were added to a 50 mL flame dried round bottom flask in a N_2 atmosphere, then m-cresol (6.6 mL), isoquinoline (0.1 mL), and toluene (1.3 mL) were added while stirring at room temperature (22 °C) for 2 h. The temperature was increased to 80 °C, at which the monomers became fully dissolved. Following this step, the temperature was increased in increments of 40 °C every 30 min until the temperature reached 180 °C. The reaction mixture was stirred at 180 °C for 18 h. The reaction mixture became a highly viscous brown solution. The reaction mixture was allowed to cool to room temperature (22 °C), then added dropwise to methanol, forming a brown precipitate. The precipitate was collected by gravity filtration, washed 2 times with 20 mL of hot methanol, 1 time with acetone, and then dried in the vacuum oven (60 °C, 24 h), to yield 0.694 g of the target compound (74.0 % yield). The polymerization was confirmed through FTIR, consistent with previous report.[2]

FTIR (ATR) 2964, 1716, 1677, 1582, 1495, 1486, 1443, 1313, 1245, 1194, 1106, 981 cm^{-1}.

PDI-spiro synthesis

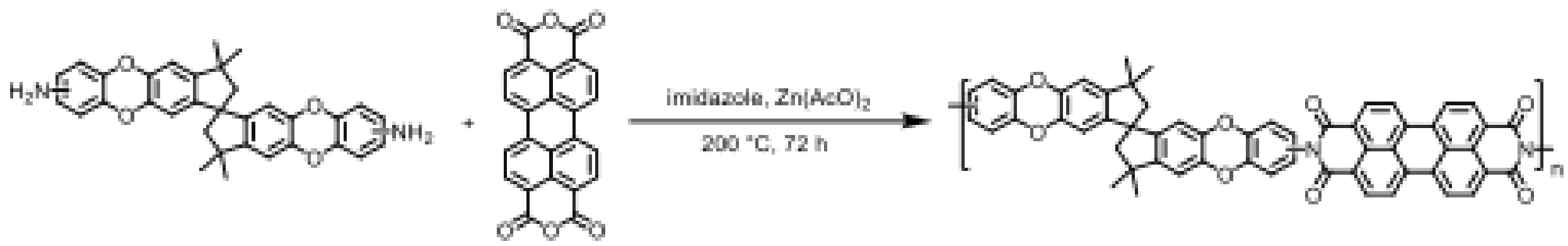

Figure S7. Scheme of synthesis of PDI-spiro.

This procedure was adapted from a previous report.[2]

SBIDA (0.50 g, 0.96 mmol, 1 equiv) and perylenetetracarboxylic dianhydride (PTCDA, 0.38 g, 0.96 mmol, 1 equiv) were added to a high-pressure tube in a nitrogen atmosphere, then imidazole (30 g) and zinc acetate ($Zn(OAc)_2$, 0.24 g) were added and stirred at 180 °C for 30 minutes until complete dissolution was achieved. The reaction mixture was then maintained at 200 °C for an additional 72 h. After completion, the mixture was cooled to 100 °C, followed by the addition of methanol to induce precipitation. The resulting solid was isolated by gravity filtration through filter paper in a plastic funnel, washed 2 times with 20 mL of methanol, and then dried in the vacuum oven (60 °C, 24 h), to yield 0.69 g of the target compound (93.0% yield). The polymerization was confirmed through FTIR, consistent with previous report.[2]

FTIR (ATR) 2952, 1708, 1662, 1595, 1508, 1485, 1366, 1357, 1318, 1251, 1175, 1106, 954 cm^{-1}.

BDI-spiro synthesis

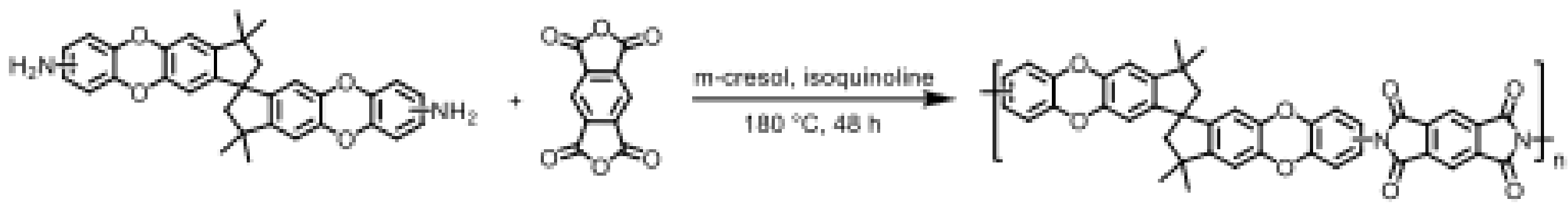

Figure S8. Scheme of synthesis of BDI-spiro.

This procedure was adapted from a previous report.[2] SBIDA (0.50 g, 0.96 mmol, 1 equiv) and pyromellitic dianhydride (PMDA, 0.21 g, 0.96 mmol, 1 equiv) were added to a 50 mL flame-dried round-bottom flask in a N_2 atmosphere, then m-cresol (8 mL), isoquinoline (0.1 mL), and toluene (1.5 mL) were added while stirring at room temperature (22 °C) for 2 h. The temperature was then increased to 80 °C after which the monomers became fully dissolved. Following this step, the temperature was increased in increments of 40 °C every 30 min until the temperature reached 180 °C, the reaction mixture was stirred at 180 °C for 18 h and turned into a highly viscous brown solution. The reaction mixture was allowed to cool to room temperature (22 °C), then it was added dropwise into methanol to form a brown precipitate. The precipitate was collected by gravity filtration, washed 2 times with 20 mL of hot methanol, 1 time with acetone, and then dried in the vacuum oven (60 °C, 24 h) to yield 0.69 g of the target compound (97.9% yield). The polymerization was confirmed through FTIR, consistent with previous report.[2]

FTIR (ATR) 2945, 1779, 1722, 1666, 1584, 1505, 1482, 1380, 1316, 1255, 1086, 864 cm^{-1}.

Synthesis of naphthopleiadene based polymer of intrinsic microporosity (NDI-tryp)

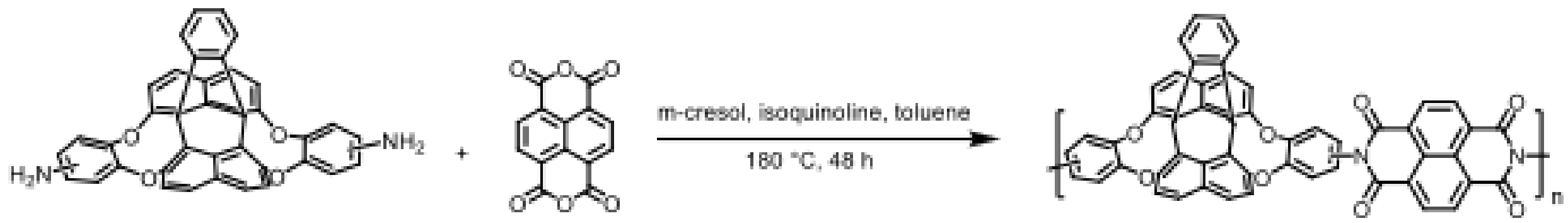

Figure S9. Scheme of synthesis of NDI-tryp.

This procedure was adapted from a previous report.[2] NPDA (0.12 g, 2.01 mmol, 1 equiv), 1,4,5,8-naphthalenetetracarboxylic dianhydride (0.05 mg, 2.01 mmol, 1 equiv) were added to a 25 mL flame-dry round bottom flask equipped with a reflux condenser in a N_2 atmosphere. To this, m-cresol (1.8 mL), isoquinoline (0.1 mL), and toluene (2.5 mL) were added and the reaction was stirred at room temperature (22 °C) for 2 h. The temperature was then increased to 80 °C after which the monomers became fully dissolved. Following this step, the temperature was increase in increments of 40 °C every 30 min until the temperature reached 180 °C and stirred for 18 h. The reaction mixture turned into a highly viscous brown solution. The reaction mixture was allowed to cool to room temperature (22 °C), then it was added dropwise into methanol, and a light-orange precipitate formed. The precipitate was collected by gravity filtration, washed 2 times with 20 mL of hot methanol, 1 time with acetone, and then dried in the vacuum oven (60 °C, 24 h) to yield 0.16 g of the target compound (90.9% yield). The polymerization was confirmed through FTIR.

FTIR (ATR) 2962, 1717, 1677, 1504, 1443, 1349, 1298, 1286, 1259, 1199, 1086, 1015 cm^{-1}.

Model Compound Synthesis

Synthesis of NDI-model

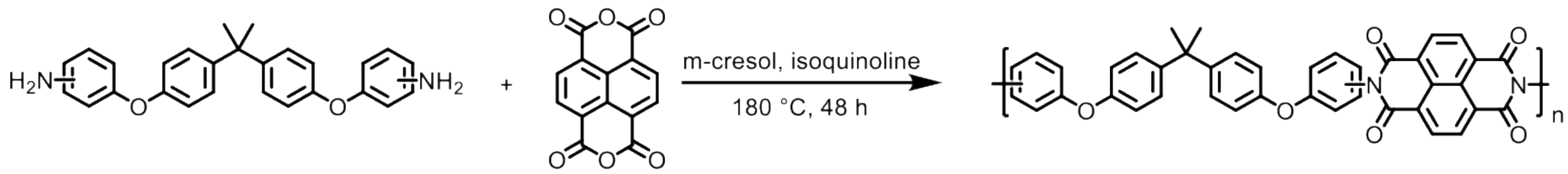

Figure S10. Scheme of synthesis of NDI-model.

This procedure was adapted from a previous report.[2]

Commercially available 4,4'-((propane-2,2-diylbis(4,1-phenylene))bis(oxy))dianiline (1.00 g, 2.44 mmol, 1 equiv) and 1,4,5,8-naphthalenetetracarboxylic dianhydride (0.65 g, 2.44 mmol, 1 equiv) were added to a flame-dry round bottom flask, then m-cresol (10 mL), isoquinoline (0.1 mL), and toluene (2 mL) were added while stirring at room temperature (22 °C), the temperature was kept for 2 h. The temperature was then increased to 80 °C after which the monomers became fully dissolved. Following this step, the temperature was increase in increments of 40 °C every 30 min until the temperature reached 180 °C, the reaction mixture was stirred at 180 °C for 18 h. The reaction mixture turned into a highly viscous brown solution. The reaction mixture was allowed to cool to room temperature (22 °C), then the reaction mixture was dropwise added into methanol, and a brown precipitate formed. The precipitate was collected by gravity filtration through filter paper in a plastic funnel, washed 2 times with 20 mL of hot methanol, 1 time with acetone, and then dried in the vacuum oven (60 °C, 24 h) to yield 1.17 g of the target compound (71.6% yield). The polymerization was confirmed through FTIR.

FTIR (ATR) 1723, 1682, 1513, 1568, 1318, 1243, 1158, 1028 cm^{-1}.

Polymer Characterization

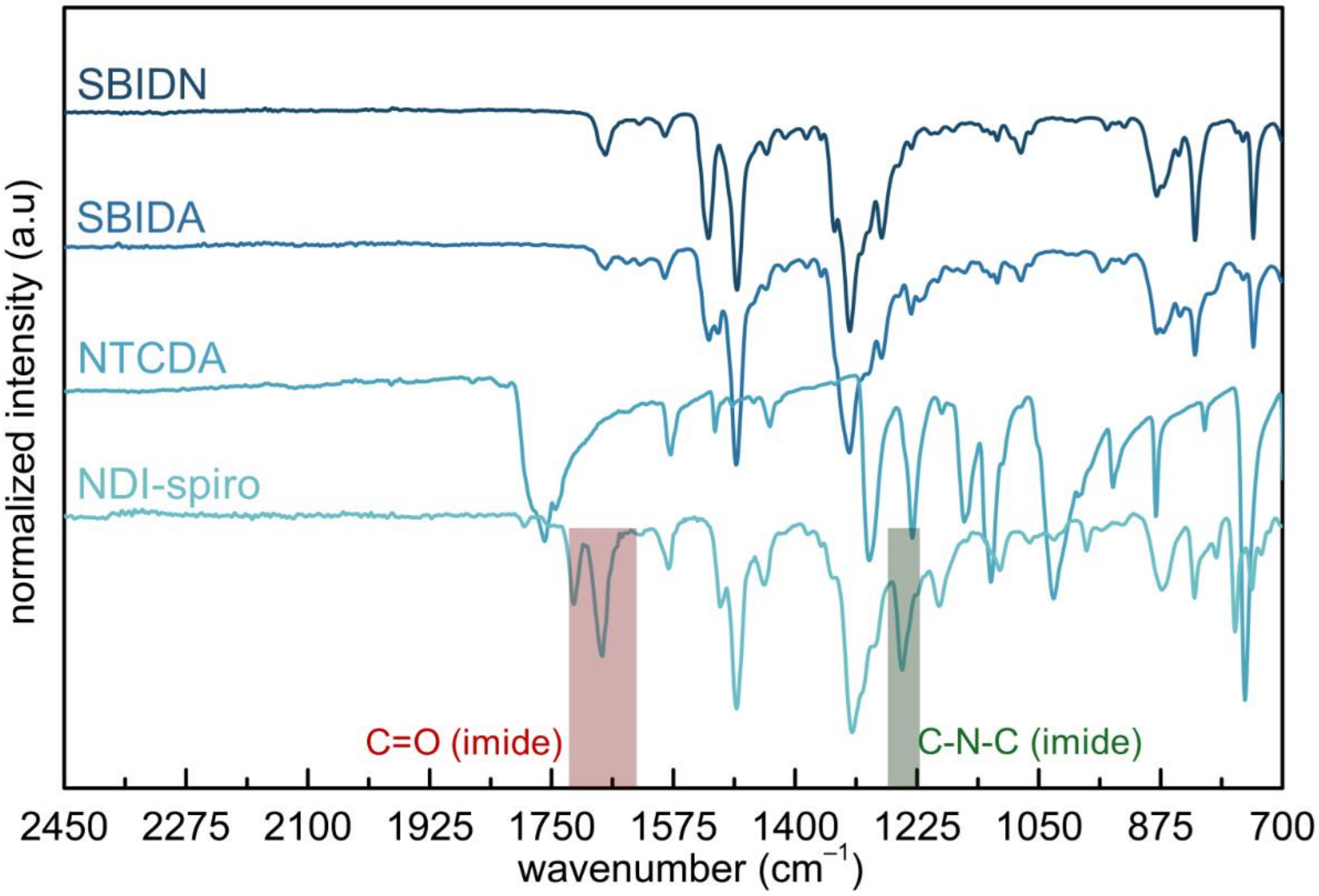

Figure S11. FTIR of NDI-spiro polymer of intrinsic microporosity, starting materials (SBIDA and NTCDA), and precursor (SBIDN).

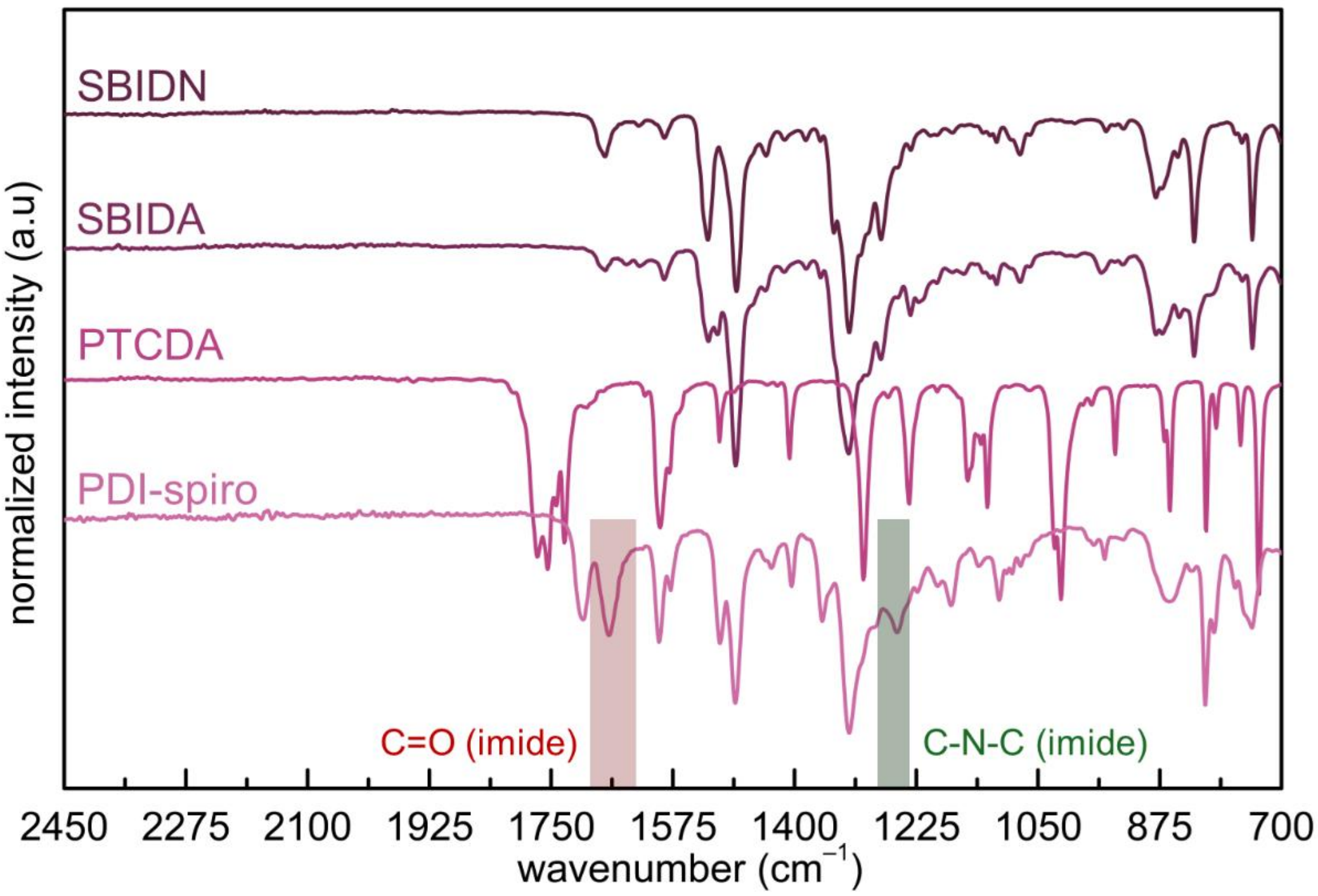

Figure S12. FTIR of PDI-spiro polymer of intrinsic microporosity, starting materials (SBIDA and PTCDA), and precursor (SBIDN).

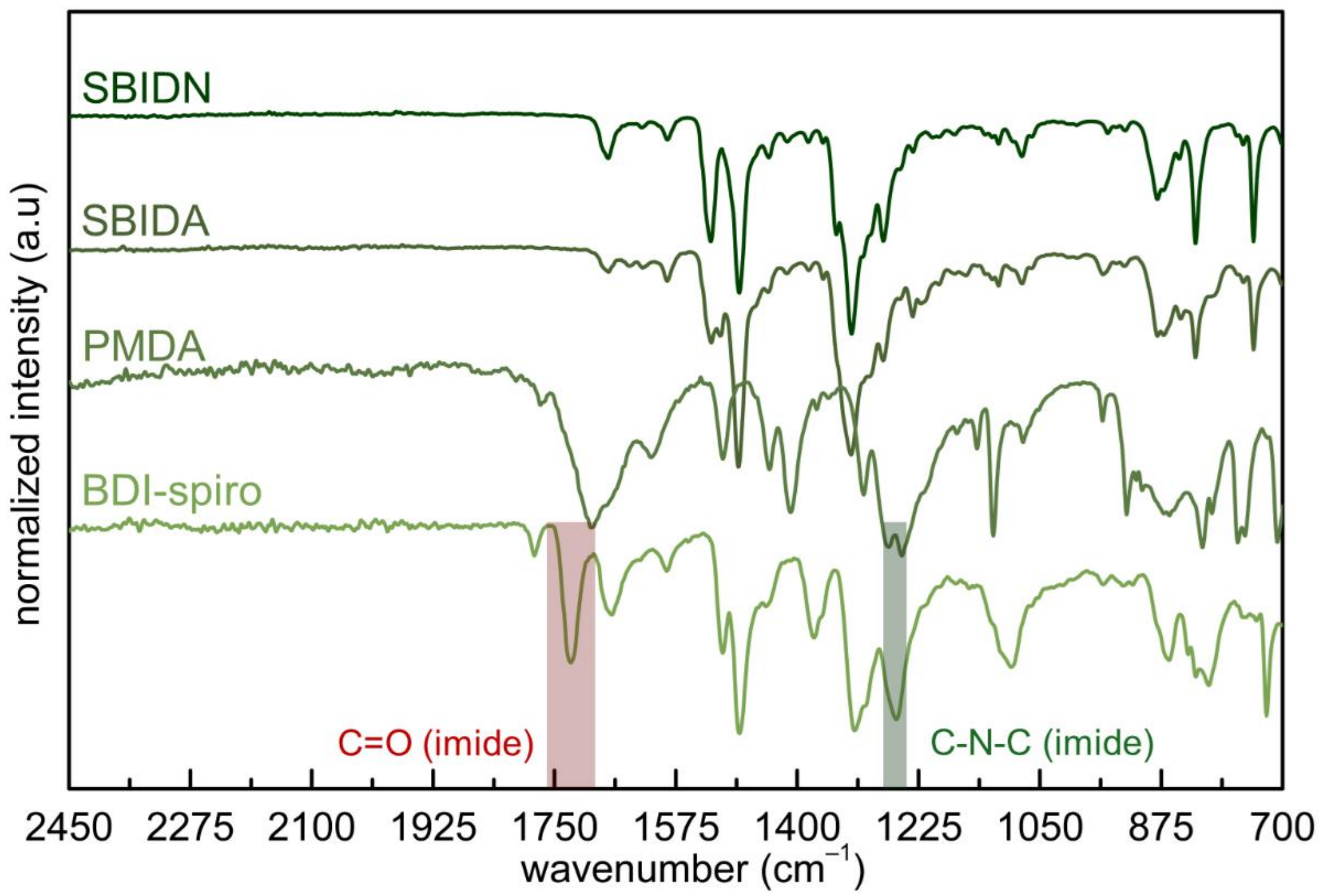

Figure S13. FTIR of BDI-spiro polymer of intrinsic microporosity, starting materials (SBIDA and PMDA), and precursor (SBIDN).

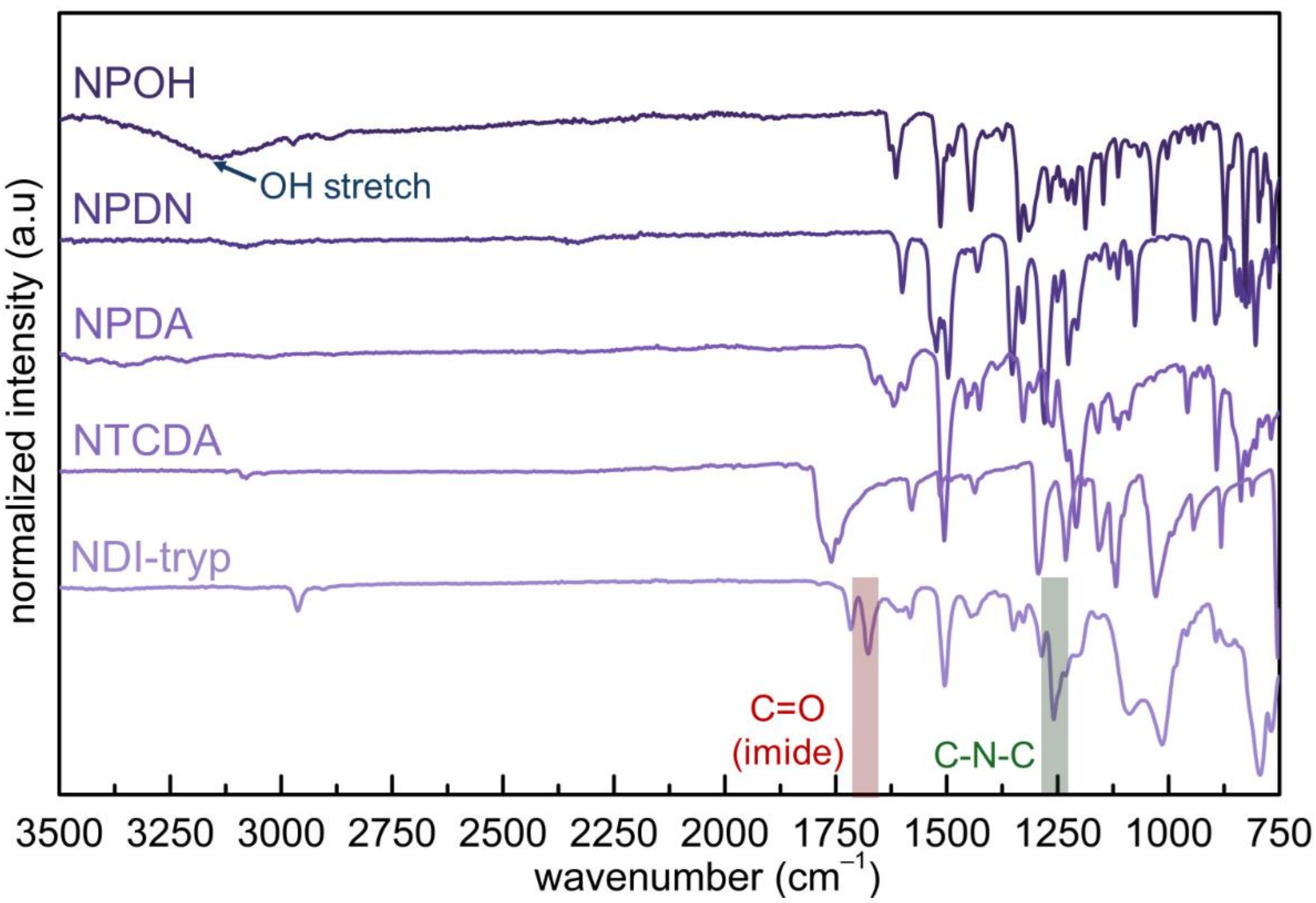

Figure S14. FTIR of NDI-tryp polymer of intrinsic microporosity, starting materials (NPDA and NTCDA), and precursors (NPDN and NPOH).

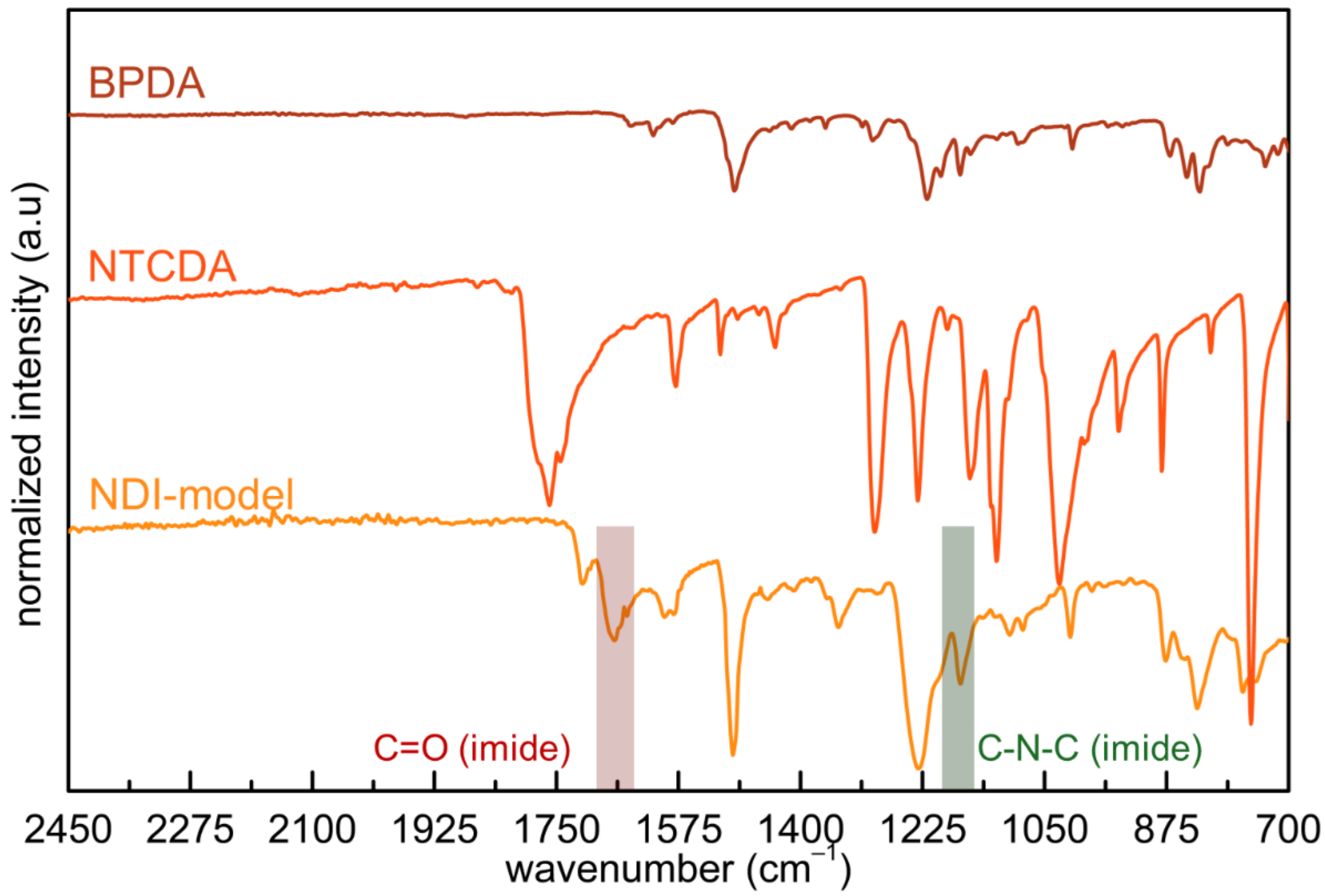

Figure S15. FTIR spectra of NDI-model polymer of intrinsic microporosity and starting materials (BPDA and NTCDA).

Electrode Preparation

Composite electrodes were prepared by mixing the active material (polymers), conductive carbon (Ketjen Black), and polyvinylidene fluoride (PVDF) in a 6:3:1 ratio in an agate mortar and pestle. Anhydrous *N*-methyl-2-pyrollidone (NMP) was added and mixed until a smooth slurry is achieved. The slurry was then cast on carbon-coated aluminum foil using a 15 µm doctor blade and dried at 50 °C for 2 h on a hot plate. The electrodes were cut with an electrode disk puncher with a diameter of 10 mm. The cast electrodes were dried at 60 °C in a vacuum oven for 24 h to remove any trace NMP or moisture.

Coin Cell Assembly

Coin cells were assembled in an argon glovebox using Li metal as the counter and reference electrode and a 1 M solution of $LiPF_6$ in ethylene carbonate:dimethyl carbonate (1:1 vol) as the electrolyte. The cathode and anode were separated by porous polypropylene separator (Celgard 2400 monolayer).

Electrochemical Measurements

Galvanostatic cycling with potential limitations

Coin cells were cycled at 2C rate for 100 cycles for cycling stability study, those were also cycled with varying cycling rates of C/10, C/5, C/2, 1C, 2C, 5C, 10C, and 20C for 10 consecutive cycles at each cycling rate and a final set of C/10 for 5 cycles, such that 1C corresponds to one discharge per hour.

The theoretical capacity calculation was obtained using the formula shown below.

$$Q_{theo} = \frac{nF}{3600 \times M}$$

Where Q_{theo} is the theoretical capacity in mAh g^{-1}, n = 2 which is the number of electrons transferred, F is the Faraday constant in C mol^{-1}, and M is the molar mass of the material in g mol^{-1}.

Cyclic voltammetry

To distinguish between surface-controlled and diffusion-controlled charge-storage mechanisms, a Trasatti analysis was performed using CV data collected at different scan rates over the same potential window for each material. The scan rates evaluated are 0.1 mV s^{-1}, 0.25 mV s^{-1}, 0.5 mV s^{-1}, 0.75 mV s^{-1}, 1.0 mV s^{-1}, 1.5 m mV s^{-1}, 2.0 mV s^{-1}, 3.0 mV s^{-1}, 4.0 mV s^{-1}, 5.0 mV s^{-1}, 6.0 mV s^{-1}, 7.0 mV s^{-1}, 8.0 mV s^{-1}, 9.0 mV s^{-1}, and 10.0 mV s^{-1} (Figure 5A, B, C and Figure 8A, B, C). For each CV, the capacitance was evaluated according to the following equation (1):

$$C = \frac{S}{2v \times \Delta U} \tag{1}$$

Where S is the integrated area of the current with respect to voltage (U), v is the scan rate and ΔU is the voltage window evaluated. The contribution from surface-controlled charge storage (C_o) was determined by plotting the calculated capacitance (C) against $v^{-1/2}$ and fitting the data linearly according to the Trasatti relation (2):

$$C(v) = av^{-\frac{1}{2}} + C_o \tag{2}$$

Where a is the slope of the linear fitting and C_o is the intercept, which represents the surface-controlled contribution to the charge-storage mechanism.

The total capacitance (C_t) was determined by plotting the reciprocal of the calculated capacitance (1/C) against $v^{-1/2}$ and fitting the data linearly according to the Trasatti relation:

$$\frac{1}{C(v)} = mv^{\frac{1}{2}} + \frac{1}{C_t}$$
(3)

Where m is the slope of the linear fitting and $1/C_t$ is the intercept, at which the total capacitance (C_t) can be calculated. The total capacitance (C_t) equals the sum of the surface-controlled (C_o) and the diffusion-controlled (C_i) contributions to the charge storage mechanism (Figure S19, Figure S20, Figure S21, Figure S22, and Figure S23).

$$C_t = C_o + C_i$$
(4)

By subtracting the surface-controlled contribution (C_o), extracted from equation (2), from the total capacitance (C_t), extracted from equation (3), the diffusion-controlled contribution (C_i) can be calculated, according to equation (4).

Electrochemical impedance spectroscopy

EIS was measured at an amplitude of 10 mV and a frequency range from 200 kHz to 100 MHz and the Nyquist plots were obtained (Figure 5, Figure 8).

To deconvolute the electrochemical processes contributing to the impedance response of the system, the Nyquist plots were analyzed with distribution or relaxation times (DRT) analysis using the freely accessible DRT tools website developed by the Wang *et al*.[5,6] The DRT method developed by Wang *et al.* transforms the impedance spectrum from the frequency domain into a distribution function of characteristic relaxation times. The total impedance of an electrochemical system is modeled according to the following equation (5):

$$Z(f) = R_\infty + i\omega L_0 + \int_{-\infty}^{+\infty} \frac{\gamma(\log\tau)}{1 + i2\pi f\tau}\, d\log\tau$$
(5)

Where R_∞ is the high-frequency ohmic resistance, ω is the angular frequency, τ is the relaxation time constant, and $\gamma(\tau)$ is the distribution of the relaxation times that describes the magnitude of electrochemical processes occurring at different characteristic timescales.

The resulting $\gamma(\tau)$ distributions were used to identify characteristic time constants associated with charge transfer, interfacial processes, and ion transport. No manual smoothing or peak fitting was applied to the DRT output (Figure S18).

Supporting Figures

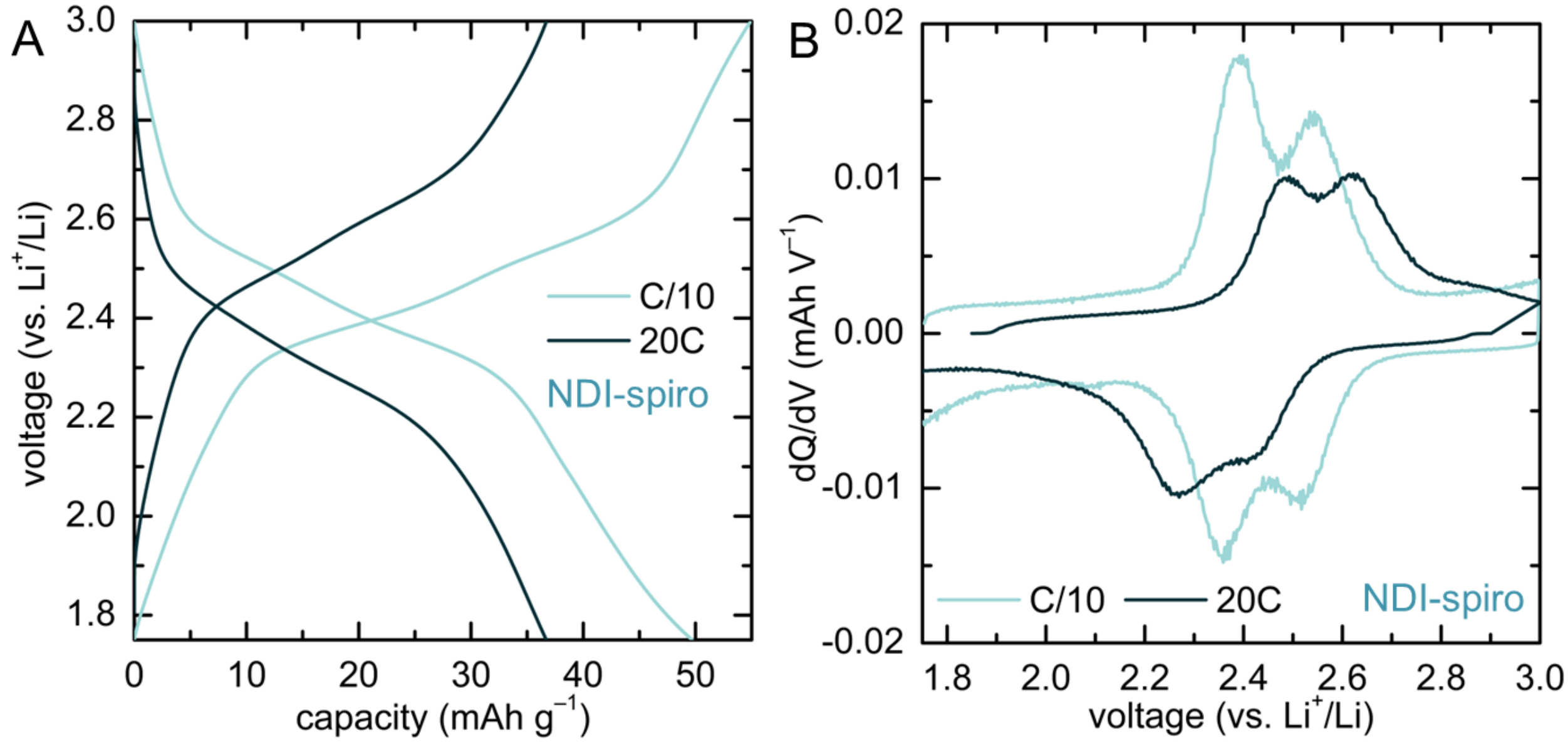

Figure S16. A. Galvanostatic cycling with potential limitations and **B.** differential capacity plot of NDI-spiro at C/10 and 20C.

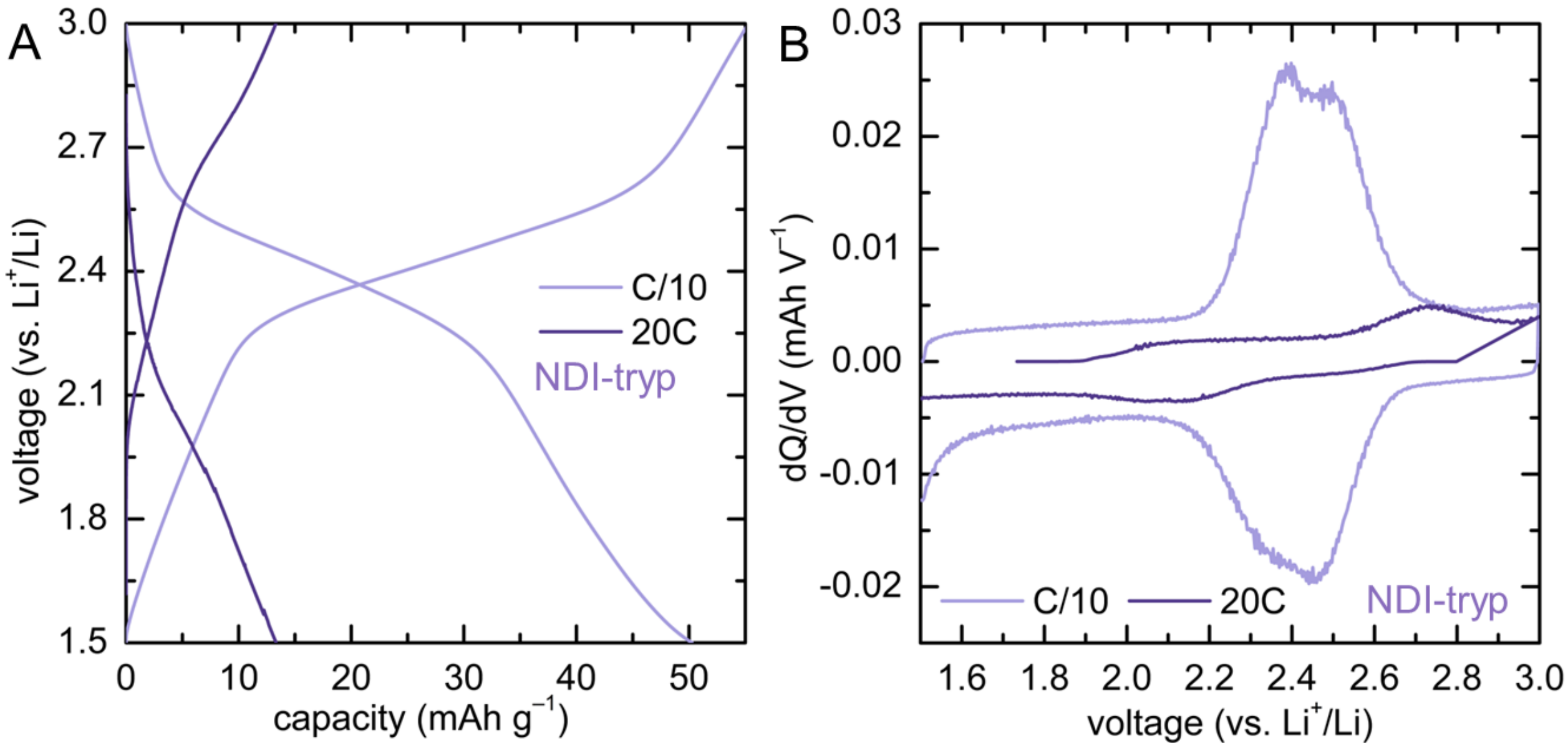

Figure S17. A. Galvanostatic cycling with potential limitations and **B.** differential capacity plot of NDI-tryp at C/10 and 20C.

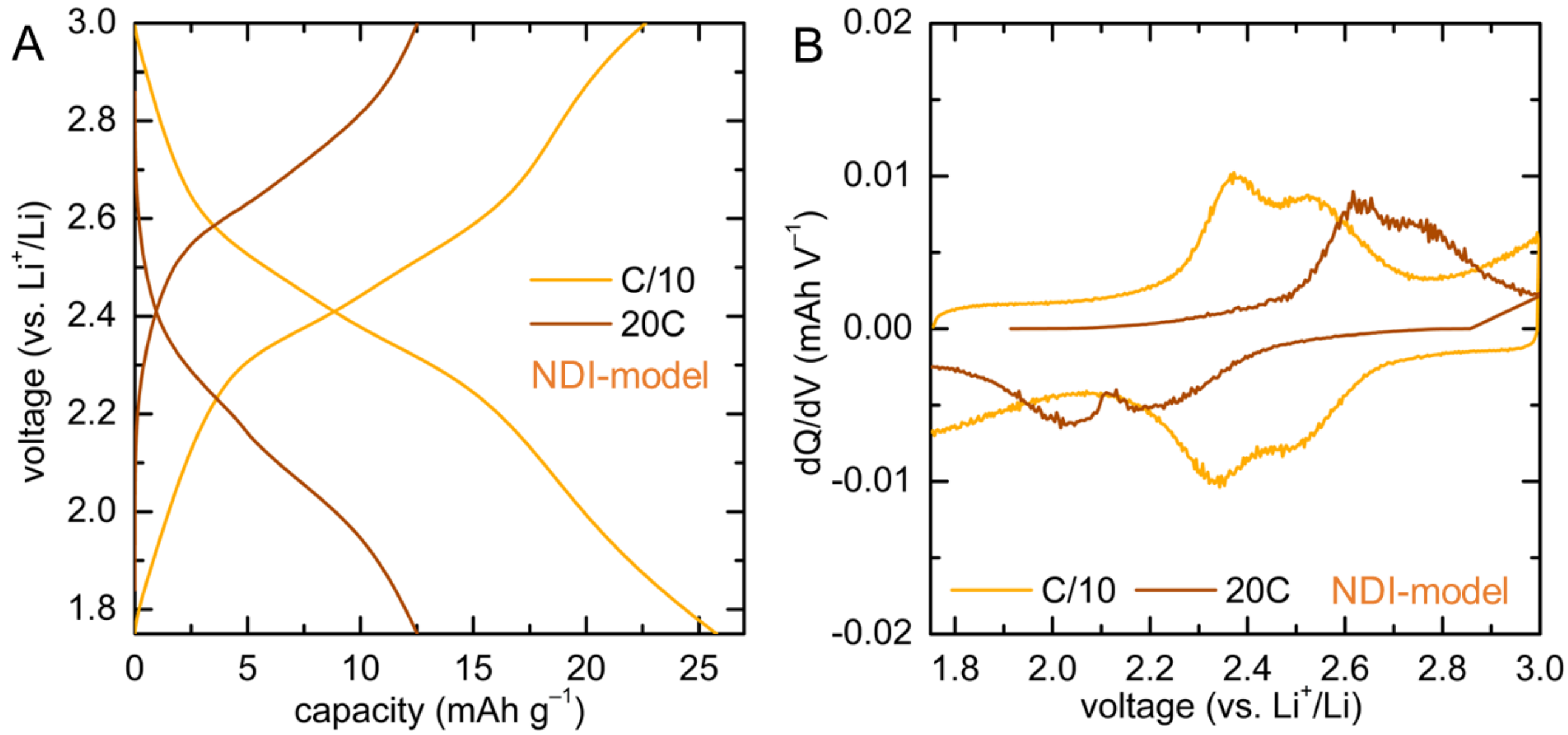

Figure S18. A. Galvanostatic cycling with potential limitations and **B.** differential capacity plot of NDI-model at C/10 and 20C.

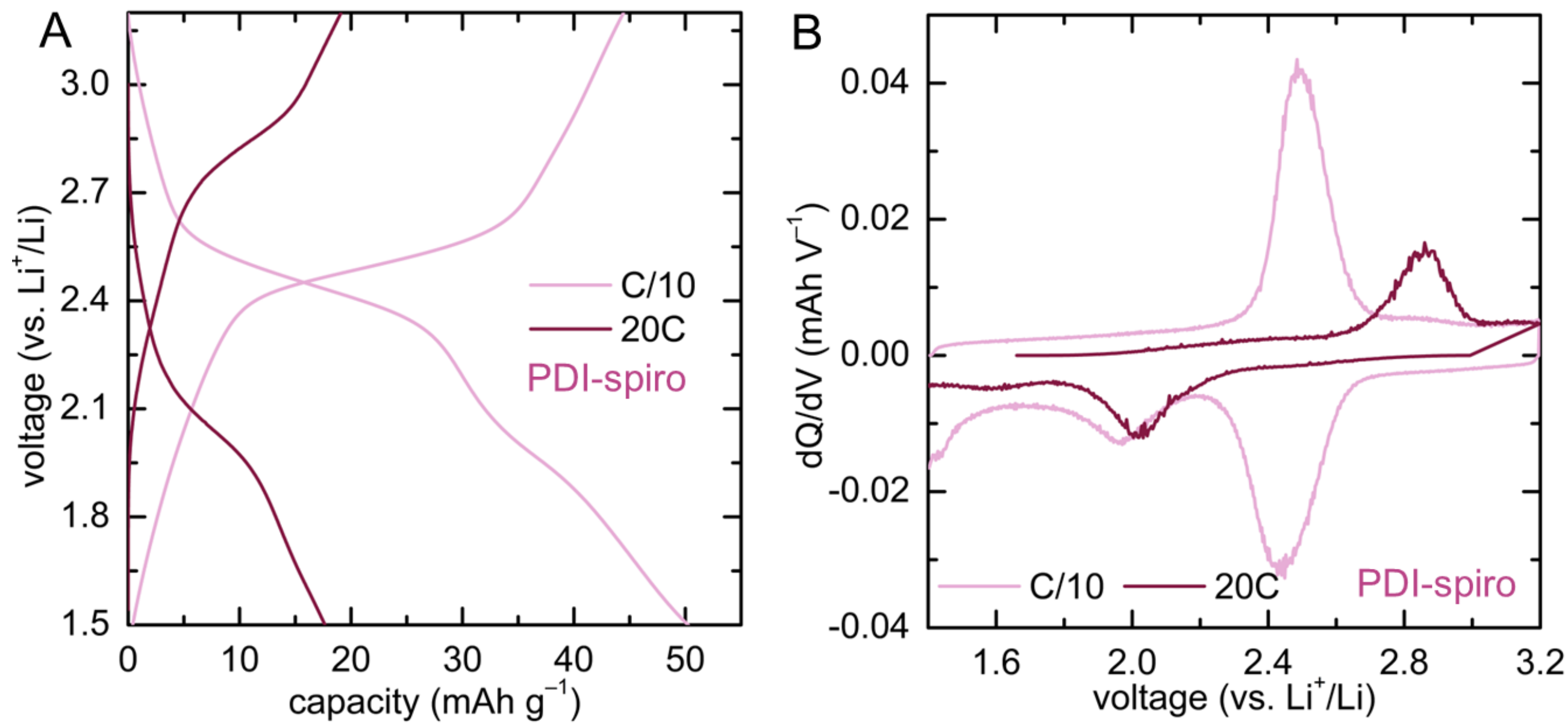

Figure S19. A. Galvanostatic cycling with potential limitations and **B.** differential capacity plot of PDI-spiro at C/10 and 20C.

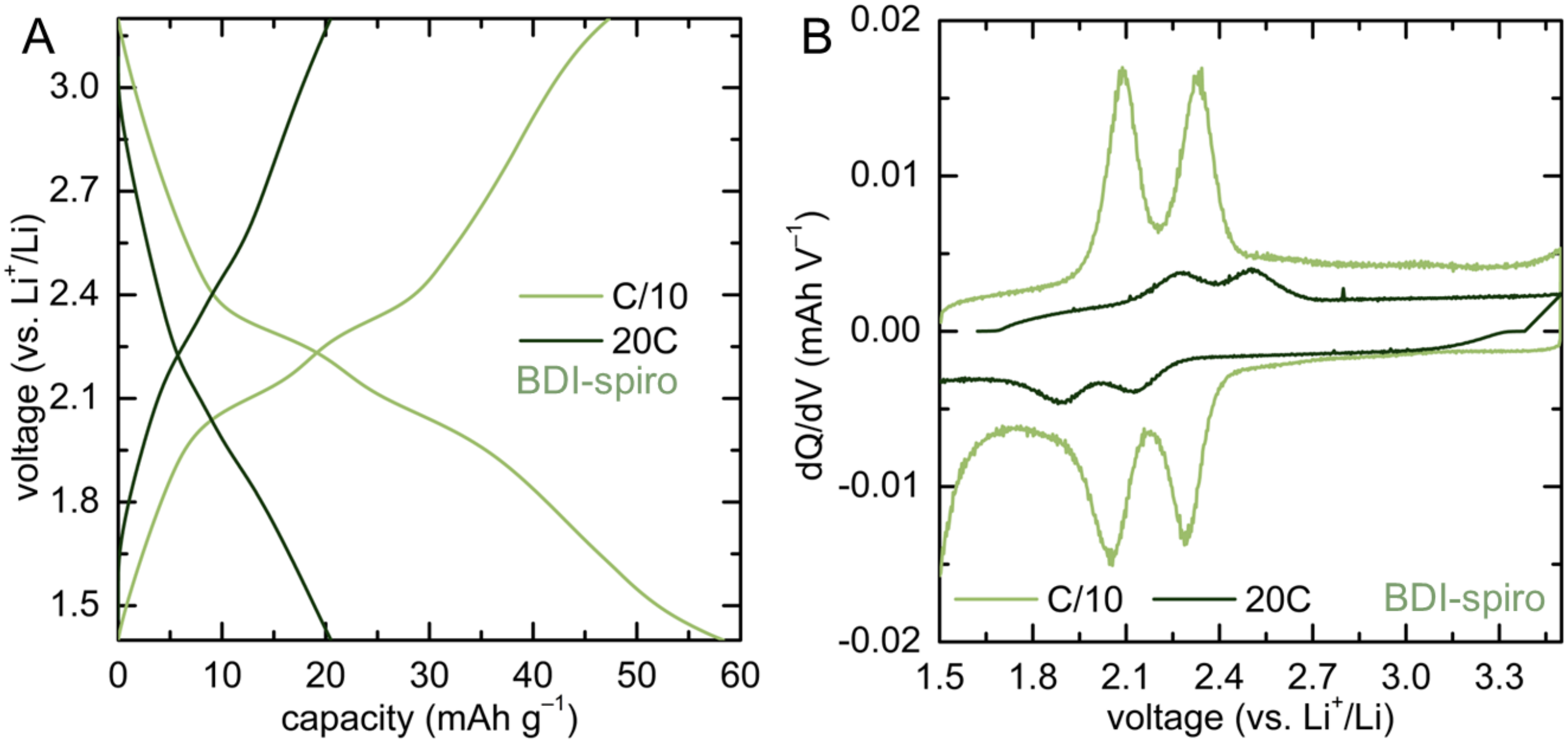

Figure S20. A. Galvanostatic cycling with potential limitations and **B.** differential capacity plot of BDI-spiro at C/10 and 20C.

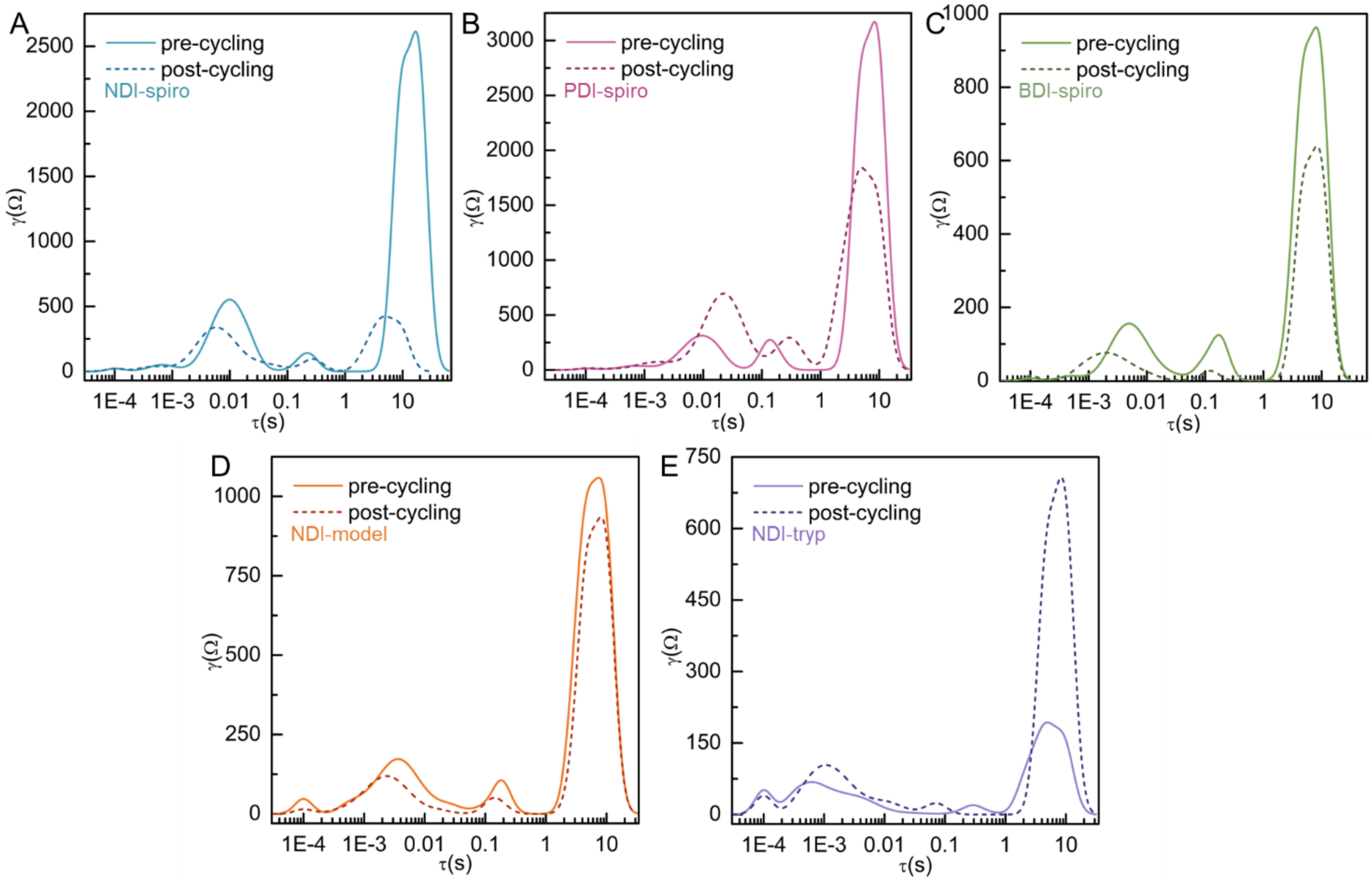

Figure S21. Distribution of relaxation times (DRT) plots for **A.** NDI-spiro, **B.** PDI-spiro, **C.** BDI-spiro, **D.** NDI-model, and **E.** NDI-tryp.

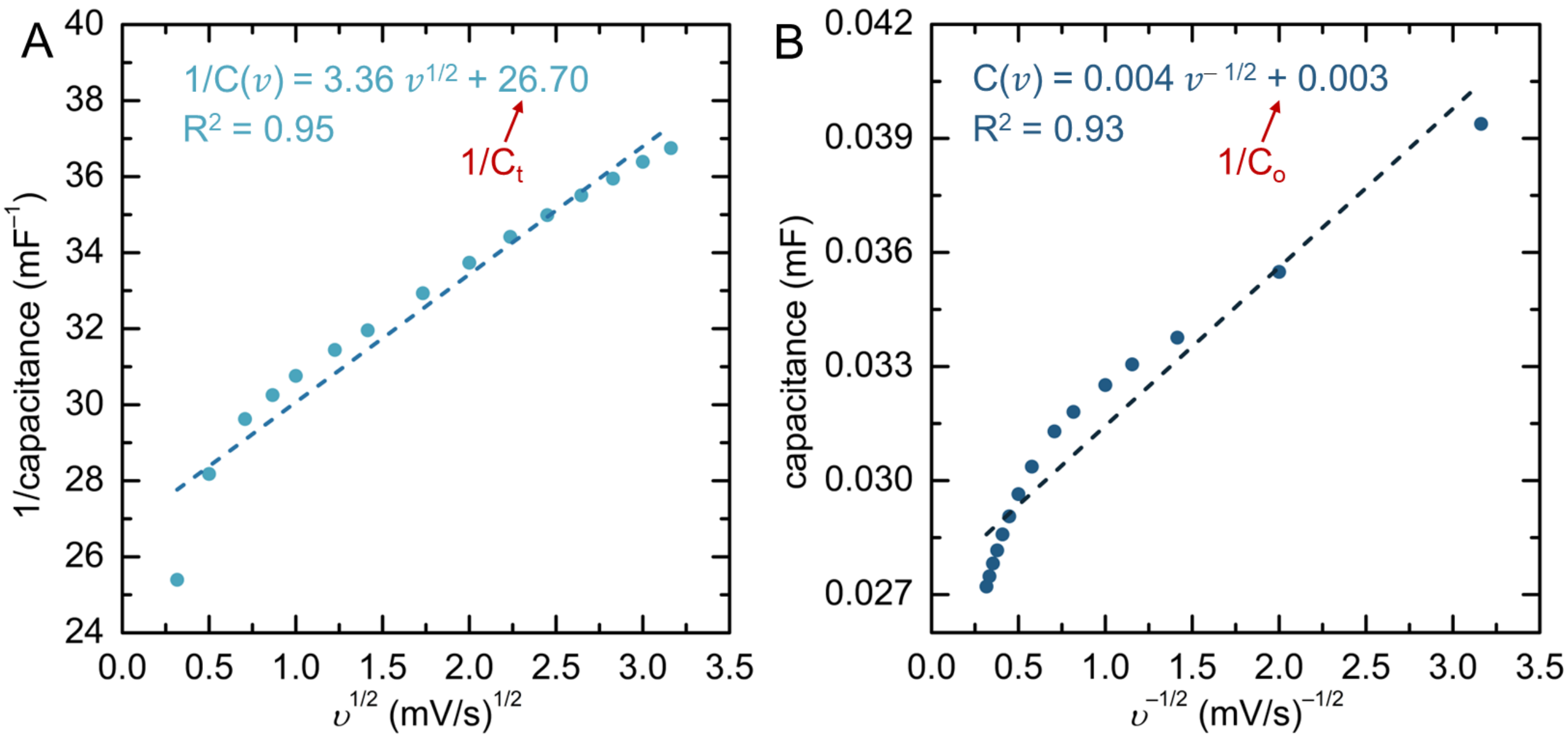

Figure S22. Linear regression used for the Trasatti analysis of NDI-spiro. **A.** Total capacitance and **B.** outer capacitance contribution.

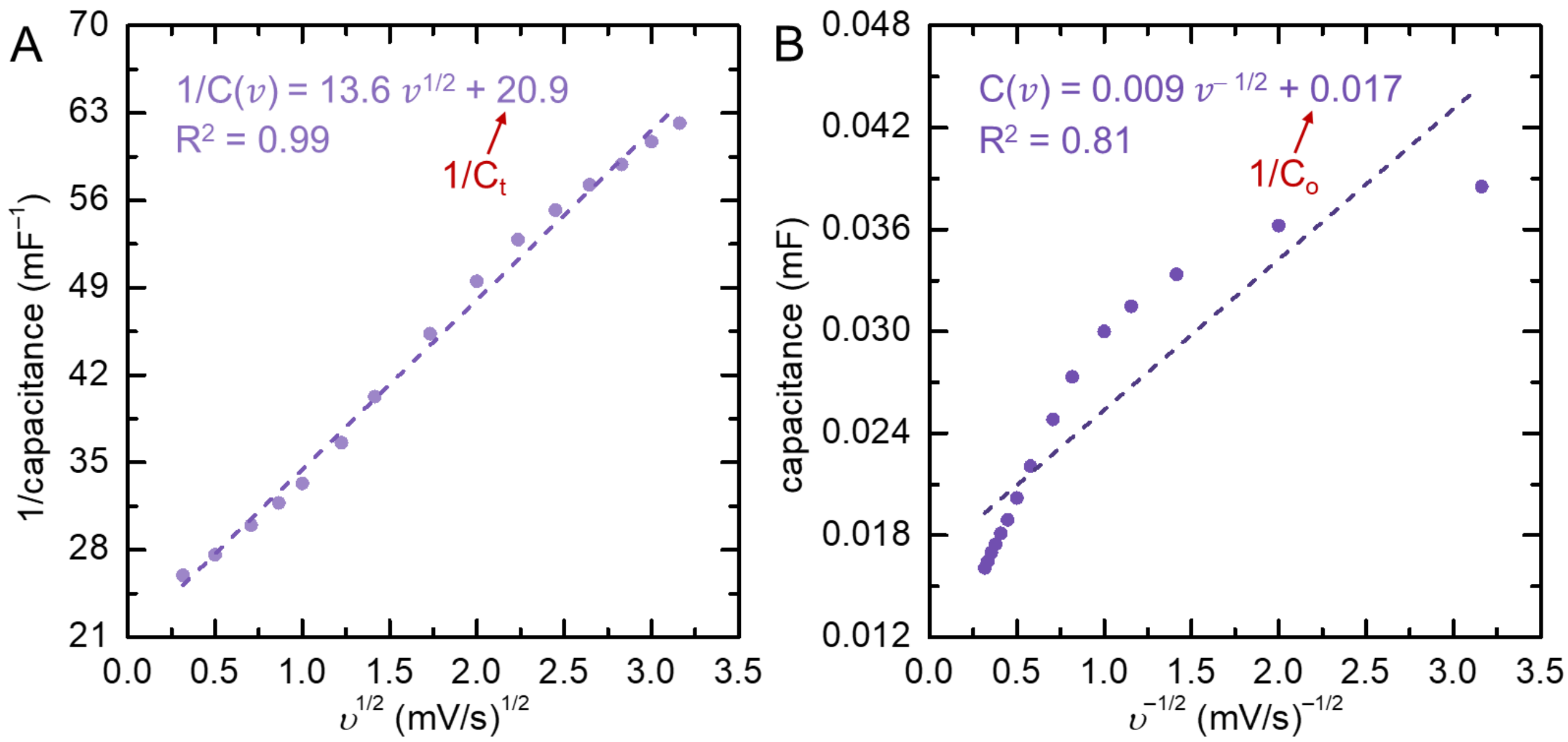

Figure S23. Linear regression used for the Trasatti analysis of NDI-tryp. **A.** Total capacitance and **B.** outer capacitance contribution.

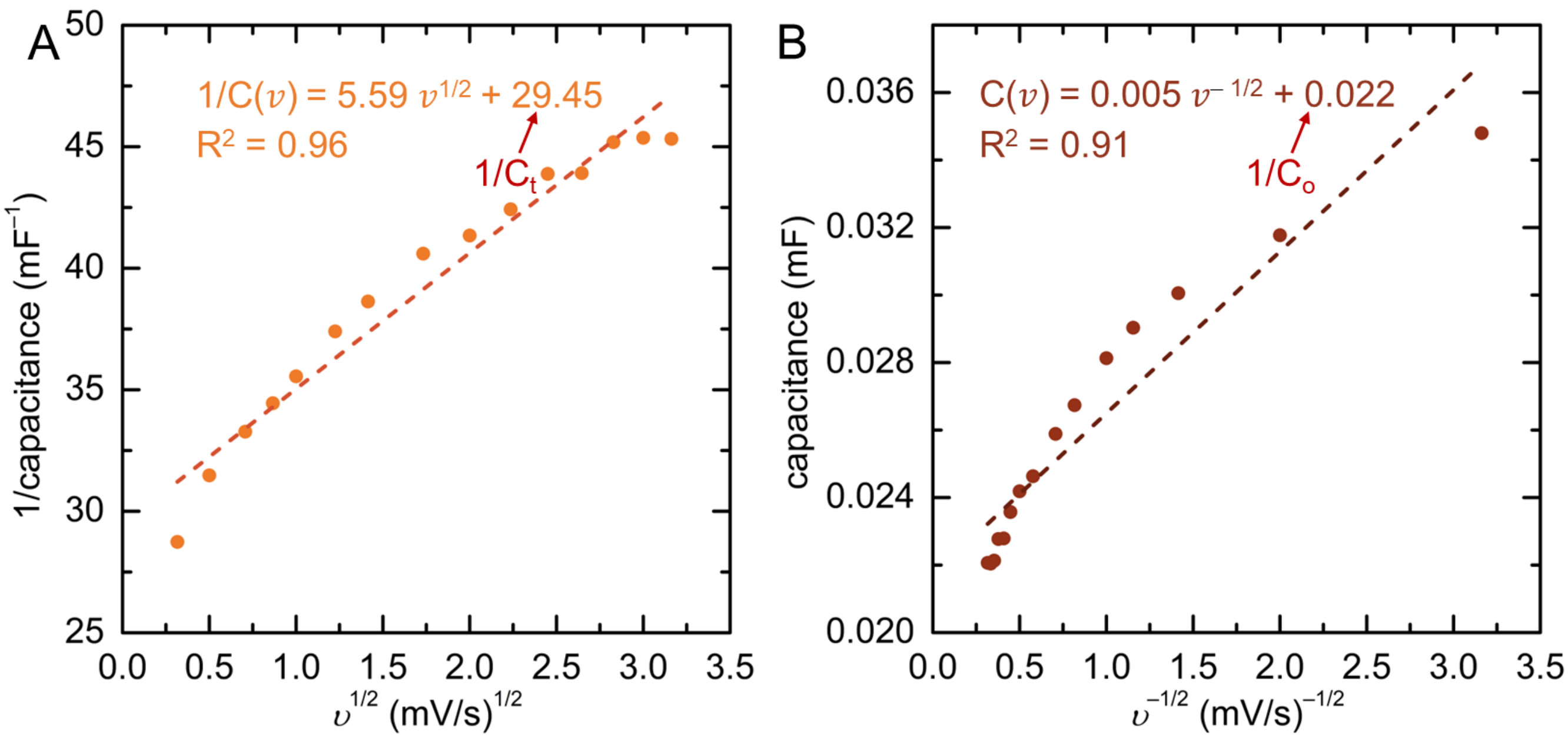

Figure S24. Linear regression used for the Trasatti analysis of NDI-model. **A.** Total capacitance and **B.** outer capacitance contribution.

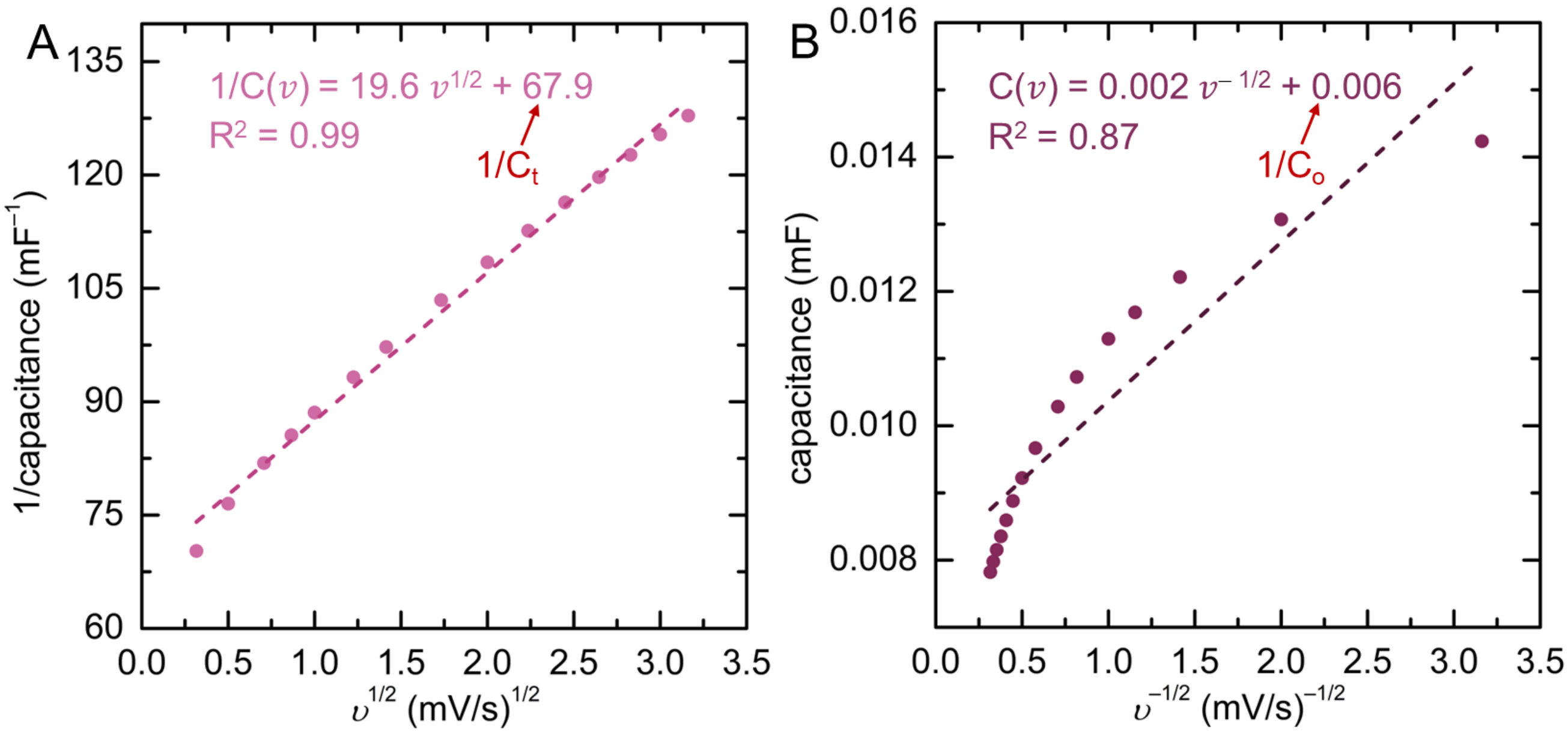

Figure S25. Linear regression used for the Trasatti analysis of PDI-spiro. **A.** Total capacitance and **B.** outer capacitance contribution.

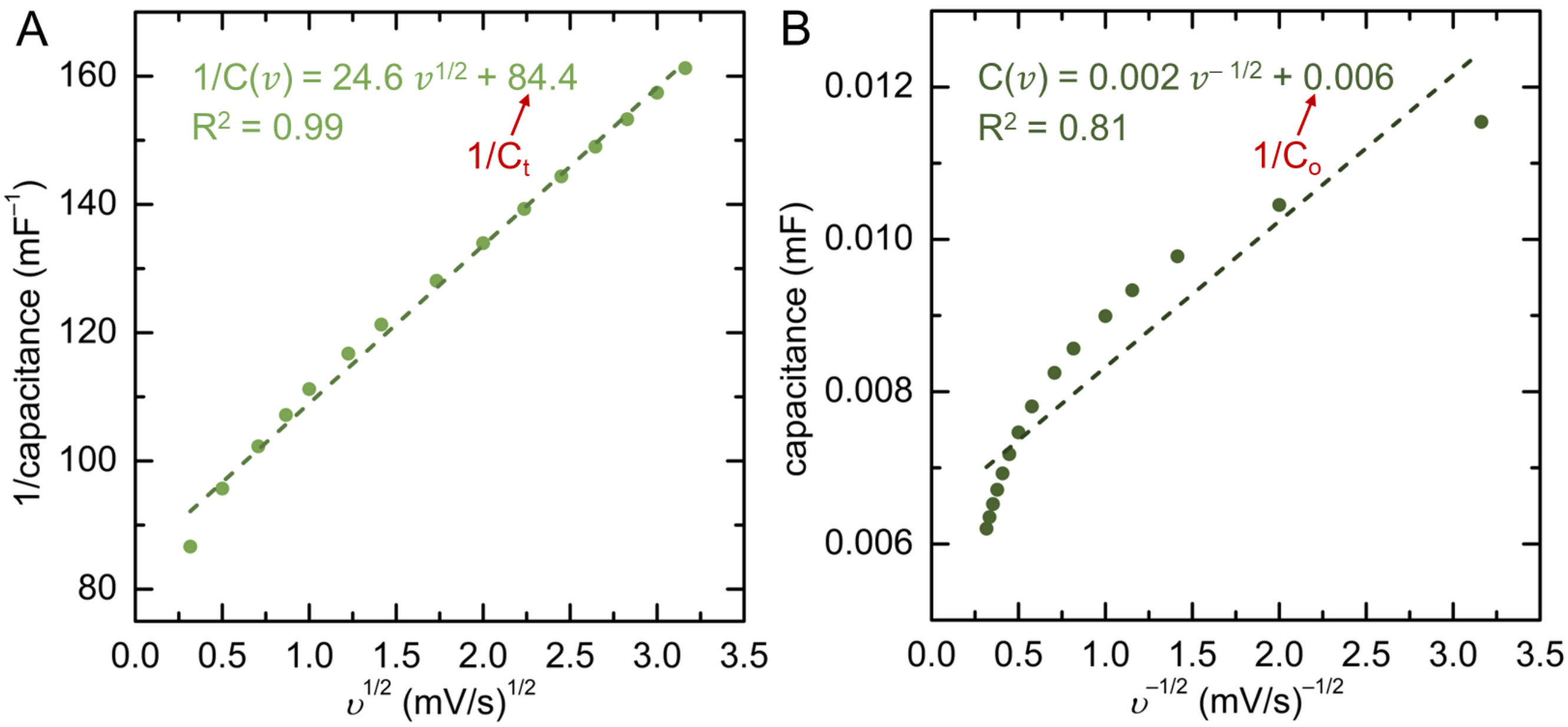

Figure S26. Linear regression used for the Trasatti analysis of BDI-spiro. **A.** Total capacitance and **B.** outer capacitance contribution.

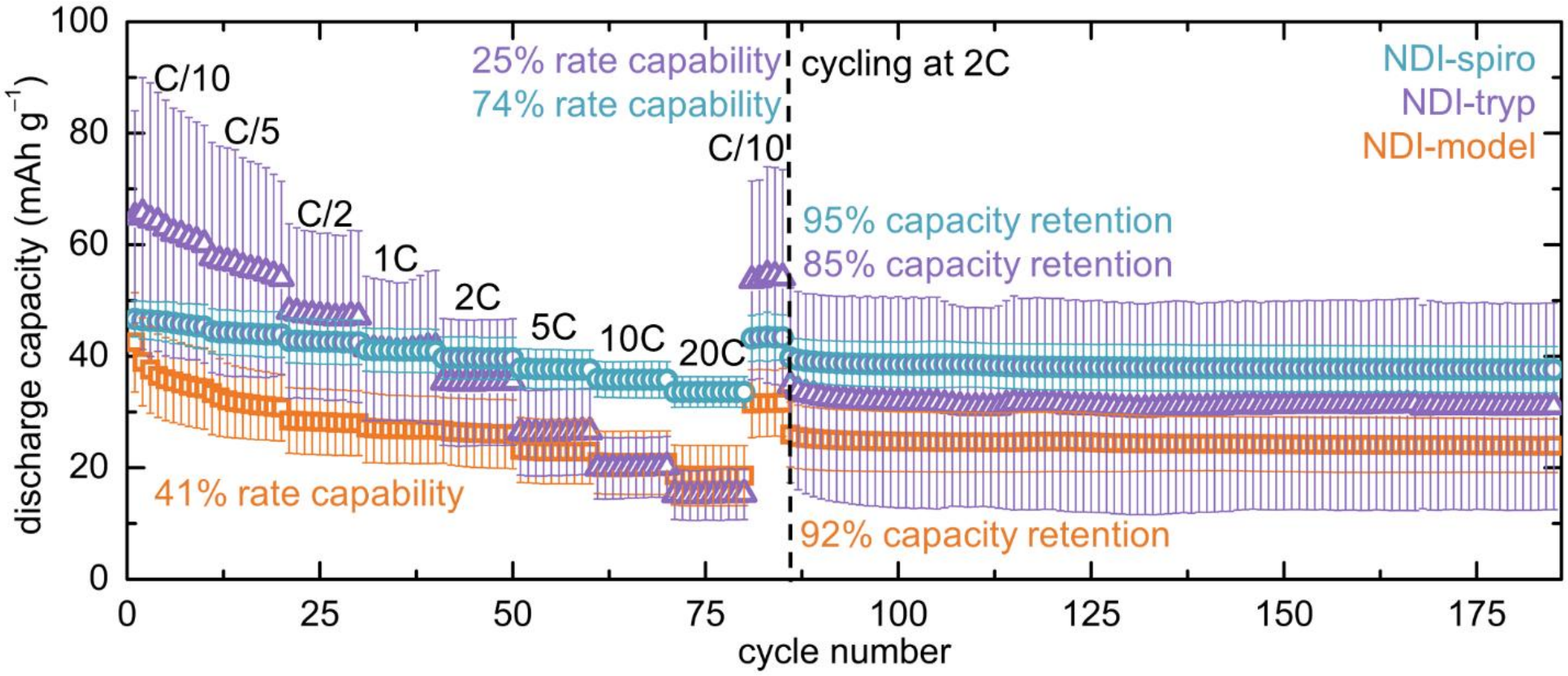

Figure S27. Average discharge capacities (left) during variable rate cycling (from C/10 to 20C) and (right) during cycling at 2C for an additional 100 cycles for NDI-spiro, NDI-tryp, and NDI-model.

The capacities at each cycle number are the average with of three representative cells for each material (symbols) with error bars representing the standard deviation from the average (lines).

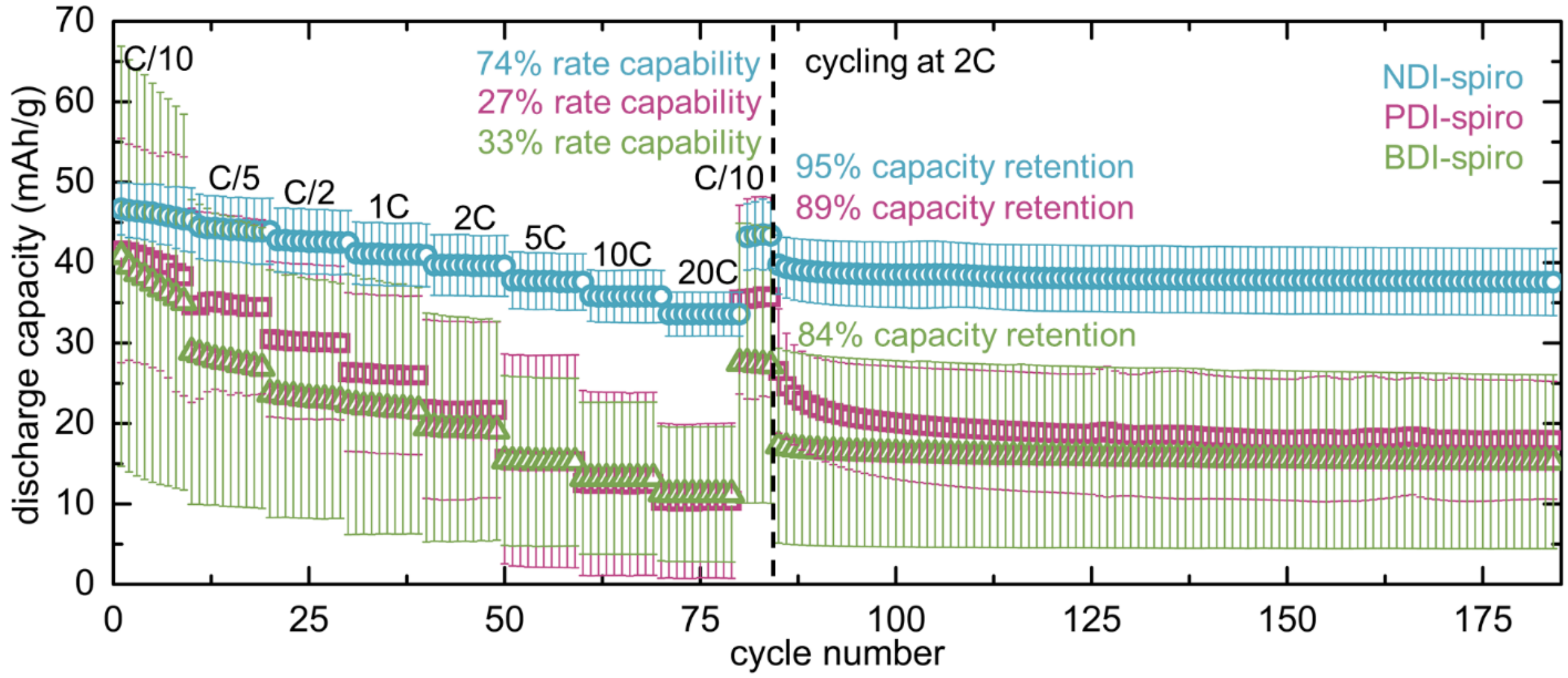

Figure S28. Average discharge capacities (left) during variable rate cycling (from C/10 to 20C) and (right) during cycling at 2C for an additional 100 cycles for NDI-spiro, PDI-spiro, and BDI-spiro.

The capacities at each cycle number are the average with of three representative cells for each material (symbols) with error bars representing the standard deviation from the average (lines).

Table S1. Data for coin cell batteries used in electrochemical measurements.

Sample	Active mass loading (mg)	Voltage range (V)	Q_{theo} (mAh g^{-1})
NDI-spiro	0.14 ± 0.05	1.75 – 3.00	71.41
NDI-tryp	0.34 ± 0.08	1.50 – 3.00	64.68
NDI-model	0.19 ± 0.06	1.75 – 3.00	83.41
PDI-spiro	0.14 ± 0.03	1.40 – 3.20	61.26
BDI-spiro	0.22 ± 0.07	1.40 – 3. 20	76.40

Table S2. Total, outer, and inner capacitance obtained by Trasatti analysis.

Sample	Total capacitance (F)	Outer capacitance (F)	Fraction of outer capacitance (% C_o)	Fraction of inner capacitance (% C_i)
NDI-spiro	0.037	0.027	72.8	27.2
NDI-tryp	0.048	0.017	34.5	65.5
NDI-model	0.034	0.022	63.9	36.1
PDI-spiro	0.015	0.008	54.4	46.6
BDI-spiro	0.012	0.006	54.0	46.0

Table S3. Oxidation and reduction potentials (vs. Li^+/Li) with hysteresis from CV.

	0.1 mV s^{-1}			10 mV s^{-1}		
Sample	E_{ox} (V)	E_{red} (V)	ΔE (V)	E_{ox} (V)	E_{red} (V)	ΔE (V)
NDI-spiro	2.44	2.33	0.11	2.60	2.11	0.29
	2.57	2.49	0.08	2.70	2.33	0.37
PDI-spiro	2.53	2.43	0.10	2.71	2.27	0.44
BDI-spiro	2.12	2.03	0.09	2.23	1.82	0.41
	2.35	2.27	0.08	2.26	2.13	0.33
NDI-tryp	2.43	2.37	0.06	2.74	2.10	0.64
	2.51	2.43	0.08			
NDI-model	2.46	2.31	0.15	2.73	2.01	0.72
	2.60	2.49	0.11			

Nuclear Magnetic Resonance Spectroscopy

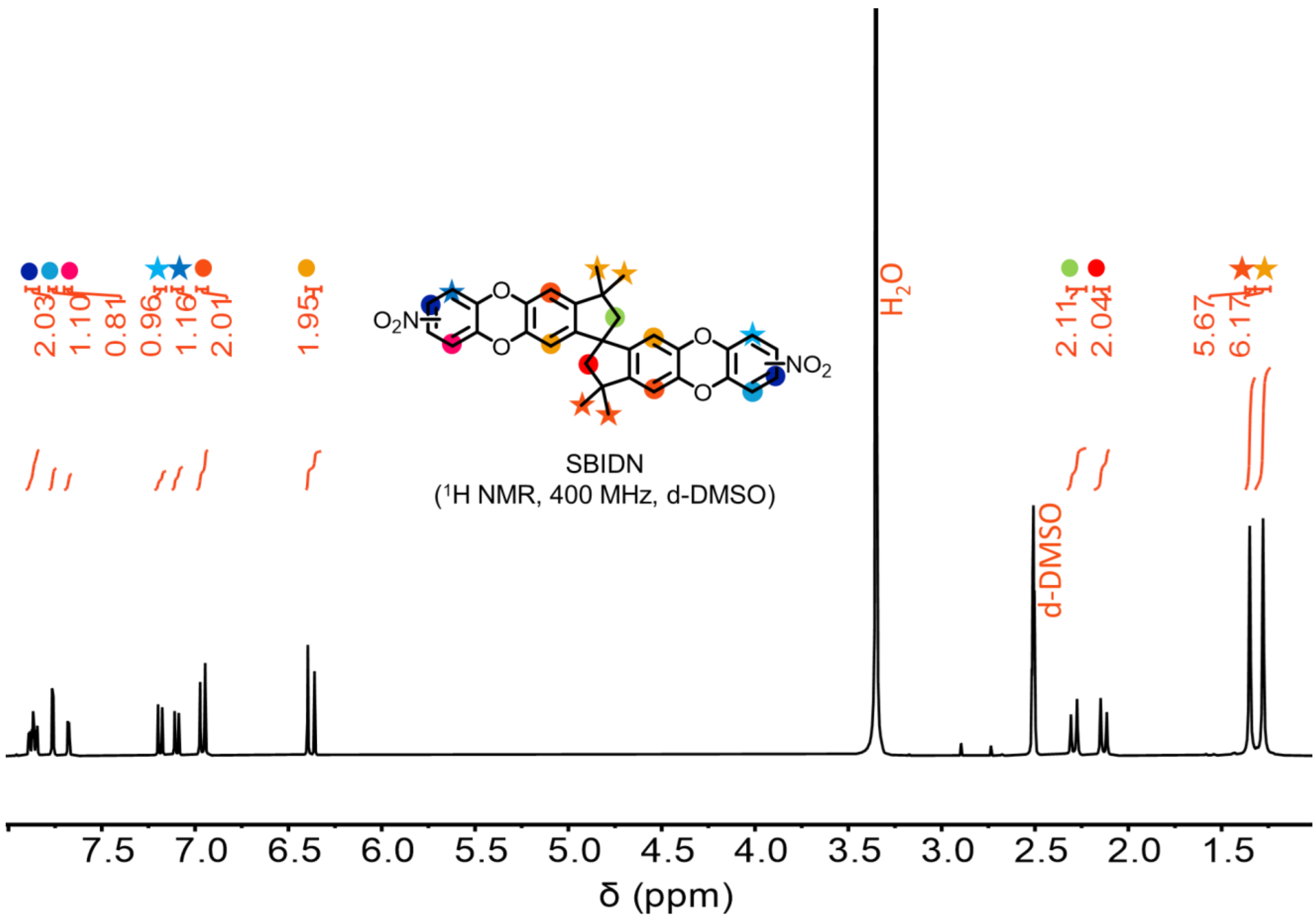

Figure S29. ^{1}H NMR (400 MHz, d-DMSO) of SBIDN.

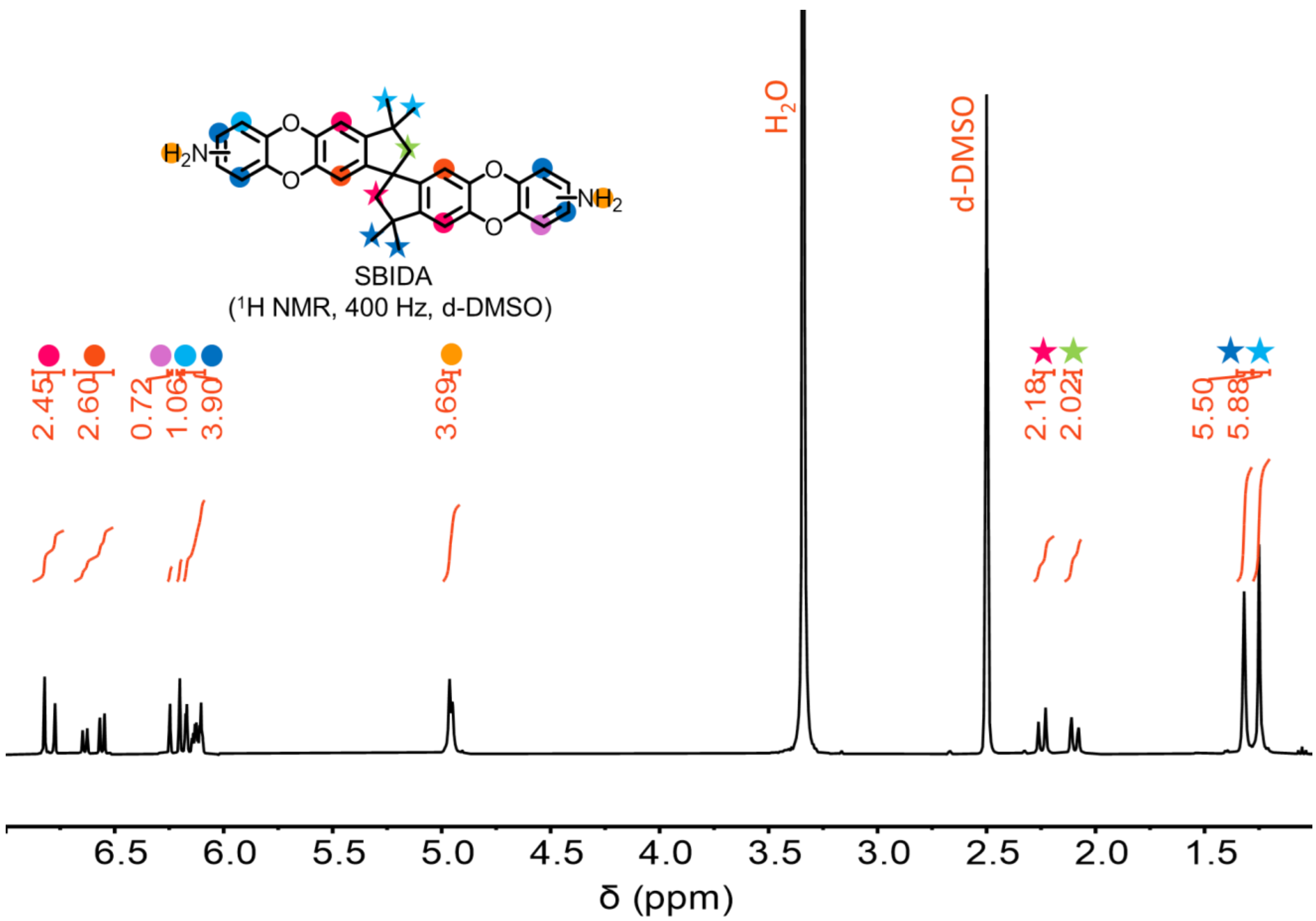

Figure S30. ^{1}H NMR (400 MHz, d-DMSO) of SBIDA.

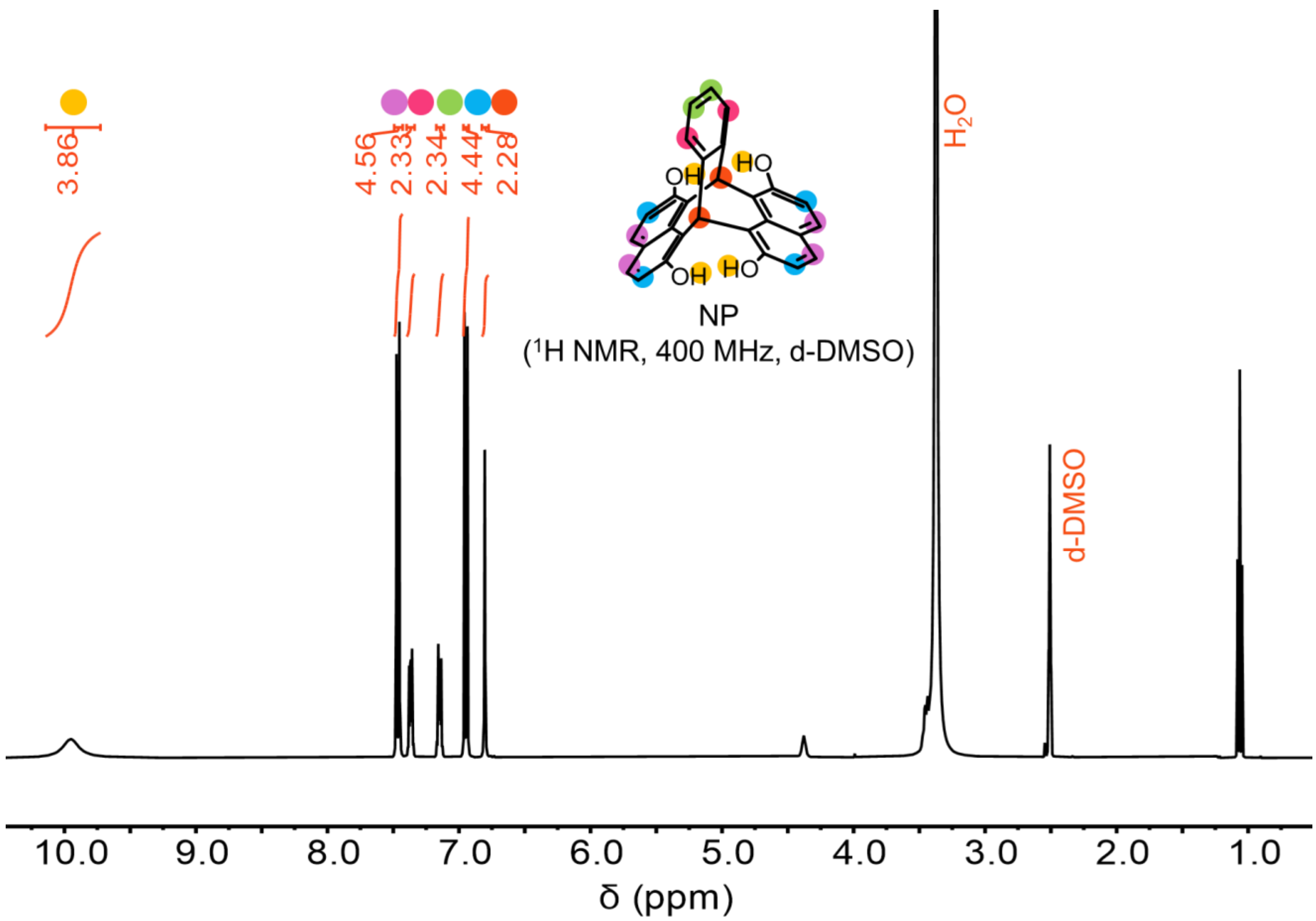

Figure S31. ^{1}H NMR (400 MHz, d-DMSO) of NP.

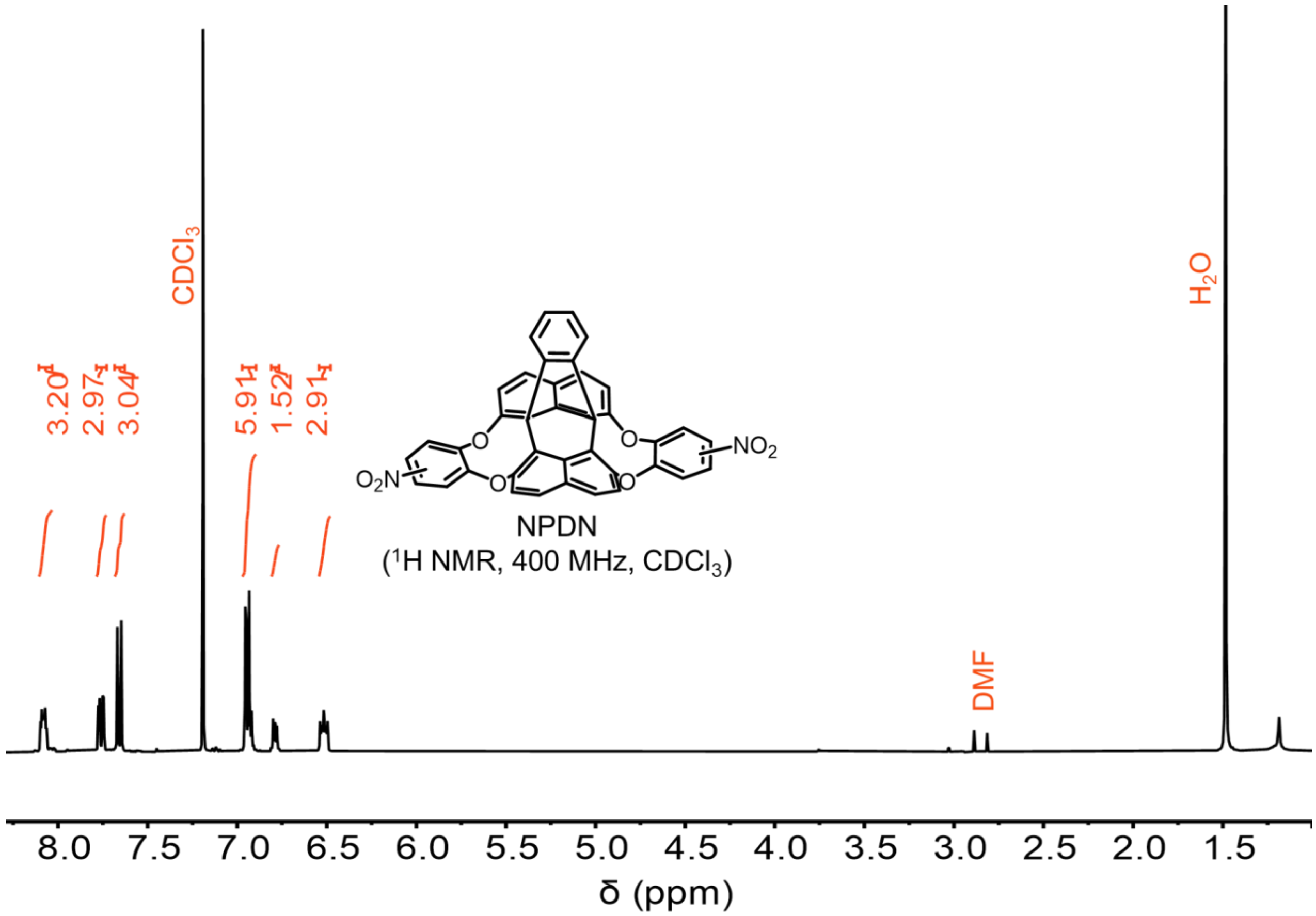

Figure S32. ^{1}H NMR (400 MHz, $CDCl_3$) of NPDN.

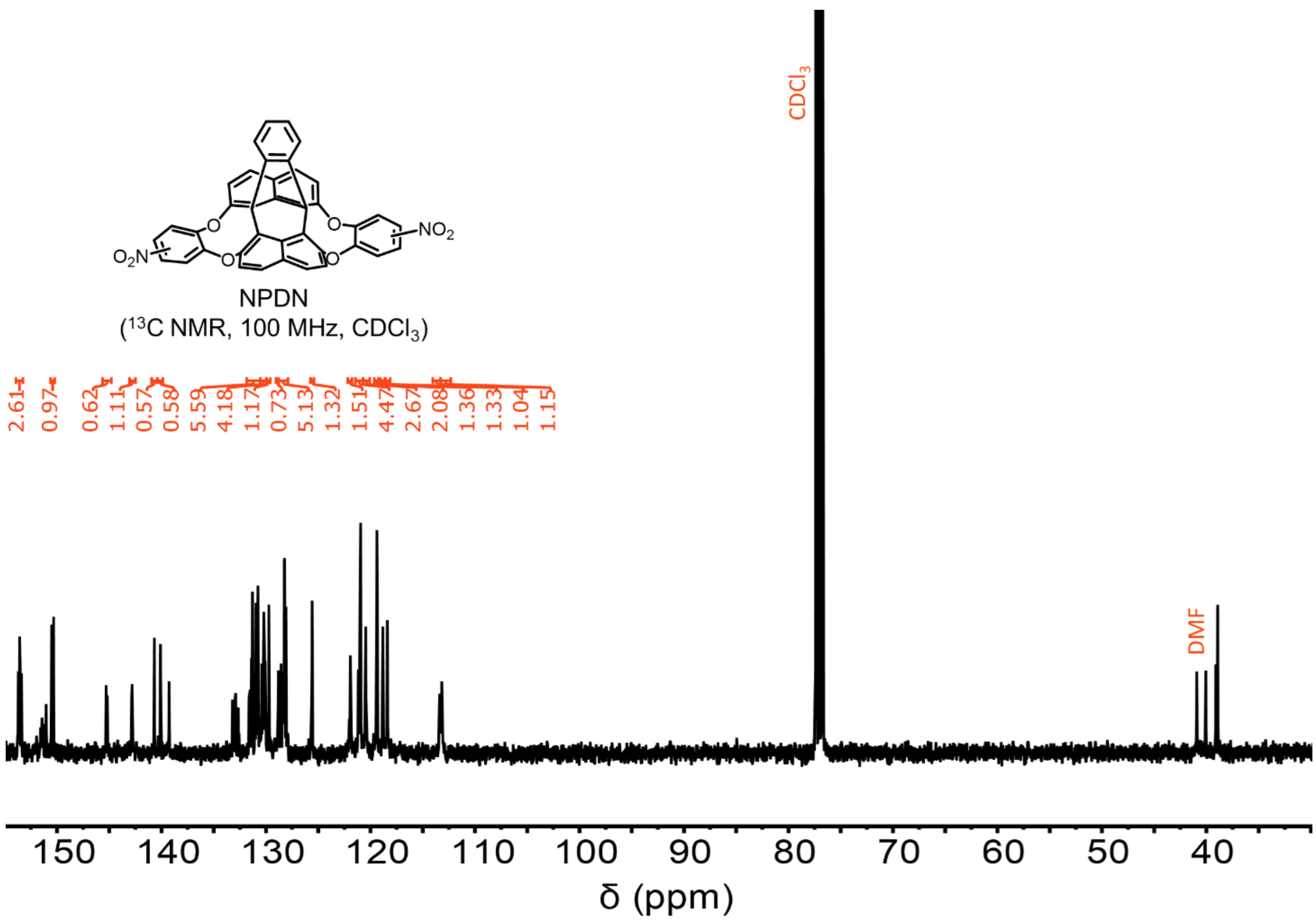

Figure S33. ^{13}C NMR (100 MHz, $CDCl_3$) of NPDN.

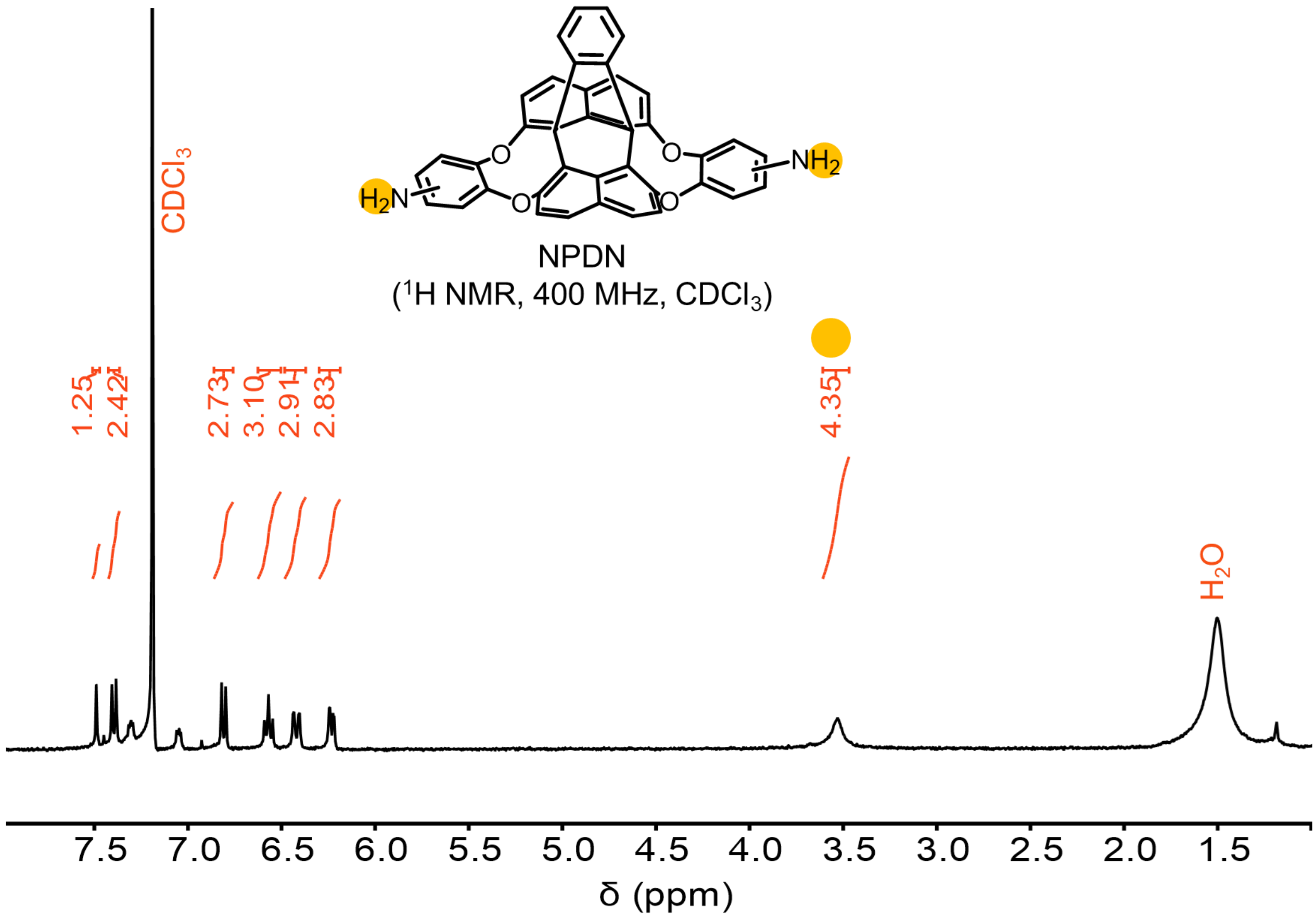

Figure S34. ^{1}H NMR (400 MHz, $CDCl_3$) of NPDA.

References

(1) Shrestha, B. B.; Wakimoto, K.; Wang, Z.; Isfahani, A. P.; Suma, T.; Sivaniah, E.; Ghalei, B. A Facile Synthesis of Contorted Spirobisindane-Diamine and Its Microporous Polyimides for Gas Separation. *RSC Adv.* **2018**, *8* (12), 6326–6330. https://doi.org/10.1039/C7RA12719G.

(2) Hasan, A. M. M.; Roy, R.; Shehab, M. K.; Davis, A. N.; Slicker, K.; Kirlikovali, K. O.; Pankow, R. M.; Farha, O. K.; Evans, A. M. Rapid Cathodic Coloration in Solution-Processable Electrochromic Polymers of Intrinsic Microporosity. *J. Am. Chem. Soc.* **2025**, *147* (19), 16331–16339. https://doi.org/10.1021/jacs.5c02014.

(3) Amin, M. K.; Ye, C.; Pang, S.; Liu, Y.; Taylor, D.; Nichol, G. S.; McKeown, N. B. Triptycene-like Naphthopleiadene as a Readily Accessible Scaffold for Supramolecular and Materials Chemistry. *Chem. Sci.* **2024**, *15* (36), 14968–14976. https://doi.org/10.1039/D4SC02755H.

(4) Wang, A.; Tan, R.; Breakwell, C.; Wei, X.; Fan, Z.; Ye, C.; Malpass-Evans, R.; Liu, T.; Zwijnenburg, M. A.; Jelfs, K. E.; McKeown, N. B.; Chen, J.; Song, Q. Solution-Processable Redox-Active Polymers of Intrinsic Microporosity for Electrochemical Energy Storage. *J. Am. Chem. Soc.* **2022**, *144* (37), 17198–17208. https://doi.org/10.1021/jacs.2c07575.

(5) Wang, Z.; Wang, Y.; Py, B.; Maradesa, A.; Liu, J.; Wan, T. H.; Saccoccio, M.; Ciucci, F. DRTtools: Freely Accessible Distribution of Relaxation Times Analysis for Electrochemical Impedance Spectroscopy. *ACS Electrochem.* **2025**, *1* (12), 2680–2689. https://doi.org/10.1021/acselectrochem.5c00334.

(6) Wan, T. H.; Saccoccio, M.; Chen, C.; Ciucci, F. Influence of the Discretization Methods on the Distribution of Relaxation Times Deconvolution: Implementing Radial Basis Functions with DRTtools. *Electrochimica Acta* **2015**, *184*, 483–499. https://doi.org/10.1016/j.electacta.2015.09.097.
